# Emergency obstetric and neonatal care availability, use, and quality: a cross-sectional study in the city of Lubumbashi, Democratic Republic of the Congo, 2011

**DOI:** 10.1186/s12884-017-1224-9

**Published:** 2017-01-19

**Authors:** Abel Mukengeshayi Ntambue, Françoise Kaj Malonga, Karen D. Cowgill, Michèle Dramaix-Wilmet, Philippe Donnen

**Affiliations:** 1 Université de Lubumbashi. École de Santé Publique : Unité d’Epidémiologie et de Santé de la mère, du nouveau-né et de l’enfant, Lubumbashi, Democratic Republic of the Congo; 2 University of Washington Tacoma, Tacoma, WA USA; 3 Université Libre de Bruxelles: École de Santé Publique: Centre de recherche en Epidémiologie, Biostatistiques et recherche clinique, Brussels, Belgium; 4 Université Libre de Bruxelles: École de Santé Publique : Centre de Recherche en Politiques et systèmes de santé-Santé internationale, Brussels, Belgium

**Keywords:** Maternal mortality, Obstetric labor complications, Perinatal death, Perinatal care, Maternal-Child Health Services, Democratic Republic of the Congo

## Abstract

**Background:**

While emergency obstetric and neonatal care (EmONC) is a proxy indicator for monitoring maternal and perinatal mortalities, in Democratic Republic of the Congo (DRC), data on this care is rarely available. In the city of Lubumbashi, the second largest in DRC with an estimated population of 1.5 million, the availability, use and quality of EmONC are not known. This study aimed to assess these elements in Lubumbashi.

**Methods:**

This cross-sectional survey was conducted in April and May 2011. Fifty-three of the 180 health facilities that provide maternity care in Lubumbashi were included in this study. Only health facilities with at least six deliveries per month over the course of 2010 were included. The availability, use and quality of EmONC at each level of the health care system were assessed according to the WHO standards.

**Results:**

The availability of EmONC in Lubumbashi falls short of WHO standards. In this study, we found one facility providing Comprehensive EmONC (CEmONC) for a catchment area of 918,819 inhabitants. Apart from the tertiary hospital (Sendwe), no other facility provided all the basic emergency obstetric and neonatal care (BEmONC) signal functions. However, all had carried out at least one of the nine signal functions during the 3 months preceding our survey: 73.6% of 53 facilities had administered parenteral antibiotics, 79.2% had systematically offered oxytocics, 39.6% had administered magnesium sulfate, 73.6% had manually evacuated placentas, 81.1% had removed retained placenta products, 54.7% had revived newborns, 35.8% had performed caesarean sections, and 47.2% had performed blood transfusions. Function 6, vaginal delivery assisted by ventouse or forceps, was performed in only two (3.8%) facilities. If this signal function was not taken into account in our assessment of EmONC availability, there would be five facilities providing CEmONC for 918,819 inhabitants, rather than one.

In 2010, all the women in the surveyed facilities with obstetric complications delivered in facilities that had carried out at least one signal function in the 3 months before our survey; 7.0% of these women delivered in the facility which provided CEmONC. Mortality due to direct obstetric causes was 3.9% in the health facility that provided CEmONC. The intrapartum mortality was also high in this facility (5.1%). None of the maternity ward managers in any of the facilities surveyed had received training on the EmONC package. Essential supplies and equipment for performing certain EmONC functions were not available in all the surveyed facilities.

**Conclusion:**

Audits of maternal and neonatal deaths and near-misses should be established and used as a basis for monitoring the quality of care in Lubumbashi. To reduce maternal and perinatal mortality, it is essential that staff skills regarding EmONC be strengthened, the availability of supplies and equipment be increased, and that care processes be standardized in all health facilities in Lubumbashi.

**Electronic supplementary material:**

The online version of this article (doi:10.1186/s12884-017-1224-9) contains supplementary material, which is available to authorized users.

## Background

Maternal mortality is a measure of a woman’s risk of dying during pregnancy, in childbirth or during the 42 days following delivery. It is a tragedy, as no woman should die giving birth [1]. In 2013, the number of maternal deaths worldwide was estimated at 292,982 [1]. In addition, there are nearly 6 million perinatal deaths (stillbirths and early neonatal deaths) that occur each year worldwide. It is in the poor countries of Africa and Asia that the risk of these deaths is highest [2].

The causes of maternal deaths are generally known [1]. Antepartum and post-partum haemorrhage, obstructed labor, severe pre-eclampsia or eclampsia, complications related to abortion, uterine rupture and postpartum sepsis are the direct obstetric complications (DOC) that account for more than 80% of maternal deaths [1, 3]. Complications from preterm births, intrapartum-related disorders or birth asphyxia, and infections are the main causes of perinatal deaths in several sub-Saharan African countries [4].

The global strategy for women’s, children’s, and adolescents’ health (2016–2030) is aligned with the Sustainable Development Goals (SDG) [5]. SDG 3.1 and 3.2 are devoted to maternal and child mortality. The targets associated with these indicators were not achieved in 2015 in the framework of the Millennium Development Goals. To achieve by 2030 the targets of SDG 3.1 – reduce global maternal mortality to fewer than 70 deaths per 100,000 live births – and SDG 3.2 – decrease newborn mortality to no more than 12 deaths per 1000 live births in all countries [6]– it is necessary to improve coverage and utilization of evidence-based interventions.

The effectiveness of emergency obstetric and neonatal care (EmONC) in reducing these mortality rates is proven [7–10]. The nine interventions constituting the EmONC services package are called EmONC signal functions. They were chosen based on their effectiveness in addressing the major causes of maternal mortality and most causes of perinatal mortality (fetal distress and respiratory distress) [10]. The availability and use of EmONC can reduce maternal mortality by 85% and perinatal mortality by more than 75%, which makes it the most effective service package for directly improving maternal and neonatal prognoses [11–14]. Nevertheless, while the effectiveness of this healthcare package is known and proven, its use and quality remain matters for concern, especially within developing countries. In Africa, where maternal and perinatal mortalities are the highest, less than 30% of women who present with obstetric complications are admitted to healthcare facilities providing EmONC [15–21].

In the Democratic Republic of the Congo (DRC), where it is estimated that 693 maternal deaths occur for every 100,000 live births [22] and 72 perinatal deaths occur for every 1000 births per year [3], we know little about the availability, use and quality of EmONC. We know that more than 70% of women give birth with skilled attendance [23]. We also know that EmONC functions should be integrated into the package of maternal-child health (MCH) care. Thus, according to national guidelines, Basic emergency obstetric and neonatal care (BEmONC) should be offered by all health facilities independent of their level in the health system, while Comprehensive emergency obstetric and neonatal care (CEmONC) constitutes a package reserved for reference facilities like reference hospitals and hospital centers [24], but at both the national and subnational levels, there is little information about the availability, use, and quality of these services.

In fact, the maternal mortality ratio (MMR) at the national level, evaluated every 5 years, gives no information about disparities between provinces or between urban and rural areas [25, 26]. The data provided by the national sanitary information system (NSIS), in addition to being frequently inconsistent, give little information about the MMR (due to underreporting of maternal deaths) or use of MCH services. They are also too lacking in completeness to be used to track progress toward reduction of maternal and perinatal mortality [27]. In terms of monitoring progress in the reduction of maternal and perinatal mortality, EmONC has the advantage of being evaluable even in small units –like the health zone (HZ) – and at short intervals, which permits the capture, even at the subnational level, of those processes which can help avoid these deaths [10]. Indicators of EmONC are key to orienting the strategies that are put in place to effectively reduce maternal and perinatal mortalities [10].

In the city of Lubumbashi, the second most populous in DRC, the availability, use and quality of EmONC services are not known [27]. This study was initiated with the aim of assessing these elements in Lubumbashi.

## Methods

### Context

In 2010, Lubumbashi’s population was estimated at 1,548,923 inhabitants in an area of 747 km^2^. Lubumbashi is divided into 11 health zones (HZ), further subdivided into 15–20 health areas [28]. The study was conducted in every HZ.

**Fig. 1:**
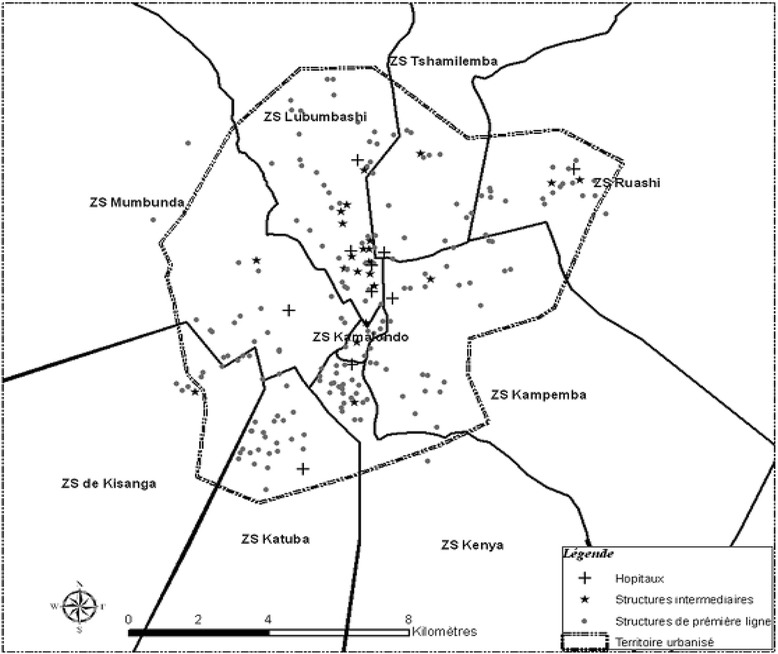
Map of health care facilities in the city of Lubumbashi, 2006 (this map from Chenge et al. [30]. Permission to publish this figure had been granted; 3770950021888; Dec 16, 2015). Only nine health zones appear on this map. The other two are a police (Kowe) and military (Vangu) camp, each contained within other health zones, and are therefore not shown

Altogether, there are more than 257 health facilities of all types in Lubumbashi, with most concentrated in urban areas (Fig. 1). Among these facilities, we distinguish the healthcare centres (HC), the general referral hospitals (GRH), the tertiary hospitals (TH: Sendwe Hospital and the Lubumbashi University Clinics) and the hospital centres (HoC). The health facilities were categorised following the descriptions supplied by the Lubumbashi health office. The HoC are polyclinics and clinics, usually private, which provide care with a technical platform similar to that seen in GRHs. There is one GRH for each HZ, with the exception of Kowe HZ, which only has one referral health centre (RHC), for a total of ten GRHs in the town of Lubumbashi. The Lubumbashi University Clinics (CUL) is the general referral hospital for the Lubumbashi HZ. Two other private hospitals, Gécamines Sud and Société Nationale Congolaise de Chemins de Fer (SNCC), fulfil this role of GRH for the Mumbunda and Tshamilemba HZs, respectively [29].

According to data from the Lubumbashi health office [28–30], 180 facilities in Lubumbashi, or more than 70%, provided maternity care in 2009. These were the facilities targeted for this study.

### Selection of the health facilities

To assess the availability, use and quality of EmONC, we selected a sample of health care facilities. The estimated minimum sample size was calculated based on the proportion of facilities providing EmONC in the DRC (5.2%) [31]. The total number of health facilities to be surveyed was 53, using an accuracy of 5% and the reported number of facilities providing maternity care (180) [29, 31].

We employed a stratified sampling, with each HZ as a stratum and the setting of facilities as a substratum. The number of facilities selected per HZ was proportional to the number of facilities in that HZ relative to the total number of facilities in Lubumbashi [32]. All the TH were automatically included in the study, as was the GRH in each HZ. For other facilities, the HZ was subdivided in two strata: urban and urbano-rural. The selection of facilities at each level was done by simple random sampling, with the pre-established list of health facilities in each substratum as the sampling frame (based on the Central Bureau of Health zone subdivisions) [29]. Thus, five health facilities were selected for each of the following HZs: Kampemba, Mumbunda, Kenya, Kisanga, Tshamilemba and Kamalondo. Six health facilities were selected for the Rwashi and Katuba HZs, eight for the Lubumbashi HZ, two for the Vangu HZ and one facility for the Kowe ZS (Additional file 1). We included only facilities with at least six deliveries per month in 2010, so four private HCs were excluded from the survey. According to the inventory carried out by the Ministry of Health – which preceded our survey – these four HCs had been in existence for less than 10 months and weren’t formally organizing maternity service activities. Consequently, they had no formal documents (registries, files, partogram, etc.) relating to maternity care offered during the period preceding the survey.

### Study design and data collection

This survey was conducted as a cross-sectional study during the months of April and May 2011, using three data collection methods. The first method, the open-ended interview, allowed us to collect data on health procedures offered that could be related to EmONC functions. The second method, the document review, allowed for data collection relating to the use of EmONC signal functions as well as on the maternal, fetal, and early neonatal outcome. The third method was observational, which allowed us to determine the availability of supplies, drugs and equipment for basic care and gave information about the management of obstetric and neonatal emergencies (Additional file 2).

The open-ended interview involved the managers of the health facilities maternity wards. The managers of the health facilities gave information on the facility’s affiliation, its location, the population it covered (population of the area or HZ), its staff profiles and the manager’s profession. For the GRHs, THs and HoCs, the head doctors were interviewed, whereas the head registered nurses answered our questions in the HC (Additional file 2).

The open-ended interview with the maternity ward managers, in addition to questions regarding their training, focused on the provision of the EmONC signal functions during the 3 months preceding the survey [10]. For this study, these are the months of January, February and March, 2011. The head of each HZ was also interviewed to validate the information obtained from the health facility managers.

Two investigators conducted the document review in the HCs, while in the GRH and HoCs and THs, four investigators were assigned to this task [10]. The document review process allowed us to collect data on the use and quality of EmONC in 2010 (births within the facility, complications of pregnancy, maternal, fetal, and neonatal deaths). To achieve this, we consulted the facilities’ records and procedure reports as well as the patients’ medical records. Not all of these sources were used in every facility. Our main data source was the procedure records. When these were missing, the patient records were searched for the necessary data. Therefore, triangulation was carried out using data from the facility records and data from the NSIS, which is provided monthly to the HZ. The quality of the records was assessed based on the presence of the items of interest to this study, and their completeness at the dates preceding our survey. Treatment rooms and delivery rooms were also inspected to assess the availability and the condition of supplies and equipment. When a pharmacy was present in the facility, the stock was checked to gauge the availability of drugs essential for the treatment of mothers and newborns [10]. Using the global positioning receiver (Garmin, Colorado 300, USA), we noted the geographic coordinates of the center of the lot of each health facility [33].

This study was approved by the medical ethics committee of the University of Lubumbashi. It also benefited from collaboration with the provincial office of the National Program for Reproductive Health (NPRH).

### Data management and analysis

The data were entered into Excel 2007 and analysed using Stata version 11.0 (College Station, TX, USA). QGIS v2.14 software allowed us to map the EmONC signal functions provided by the surveyed health facilities.

Based on the signal functions of EmONC, a facility was considered to provide basic EmONC (BEmONC) if, during the 3 months preceding the survey, it had carried out EmONC signal functions 1–7. It was defined as having comprehensive EmONC (CEmONC) if it had carried out functions 1–9 [10].

The availability, use and quality of EmONC were assessed according to WHO standards [10]. WHO definitions were used to identify direct obstetric complications (DOC; ante- and post-partum haemorrhage, severe pre-eclampsia or eclampsia, obstructed labour, complications relating to abortion, ectopic pregnancy and postpartum sepsis) and indirect obstetric complications (IOC: anaemia, malaria and AIDS) [10].

To extrapolate these observations to all facilities in Lubumbashi, the data describing the availability, use and quality of EmONC in the surveyed facilities was divided by an estimation factor. This factor was set at 0.5932, which corresponds to the proportion of births that took place in the surveyed facilities relative to those expected in Lubumbashi in 2010 [10]. Given that all the reference facilities were included in our study sample, extrapolation applied only to the HCs and HoCs. The 95% confidence intervals (95% CI) were calculated for all the estimated values for the city of Lubumbashi.

Concerning data analysis, descriptive statistics (means, standard deviations, medians and proportions) were used to describe the profile of the surveyed health facility managers. Proportions were used to assess the availability and use of the various EmONC signal functions [32].

## Results

### Profile of the health facilities

A total of 53 health facilities was selected to assess the availability, use and quality of EmONC in Lubumbashi. Among these facilities, 33 (62.3%) were HCs, nine (17.0%), were GRHs, nine were HoCs, while two (3.8%) were THs. The majority (79.2%) of these facilities were managed by men. These facilities were located in urban (41 facilities), urban–rural (11 facilities) and rural areas (one facility). Health facilities that provided maternity services were predominantly private (32.1% were owned by individuals, 20.7% by religious organisations, 13.2% by non-governmental organizations, and 7.6% by private companies); only 26.4% of the selected health facilities were public.

Health facility managers were doctors (41.5%), nurses (22.6%) or administrative executives (9.4%); 26.5% of facilities were run by a non-healthcare professional (priest, nun, pastor). The median tenure of each manager was 9 years (minimum 1, maximum 30). Most (83.0%) maternity ward managers in the surveyed facilities were women, either nurses (69.7%) or midwives (30.3%). Their median tenure in the maternity ward was 7 years (minimum 1, maximum 9).

In these facilities, none of the maternity ward managers had received training on the EmONC package according to the directives issued at the national level. Overall, only half of the managers (50.9%) had been trained in the use of a partogram and trained to give essential care to the newborn. A small proportion of the maternity ward managers reported having received training for elements of EmONC: the active management of the third stage of labour (AMTSL, 43.4%), the safe use of blood transfusion (26.4%), the management of pre-eclampsia or eclampsia (28.3%), the use of instruments to deliver the fetus (15.1%), the management of neonatal asphyxia (35.8%) and the management of severe postpartum or neonatal sepsis (28.3%).

### Staff present in obstetric settings

The surveyed health facilities employed a diverse mix of staff in their maternity wards. At the THs, CUL and Sendwe, and in the GRHs, medical doctors represented the largest category of staff (36.0% to 37.6%), whereas the majority of the staff in the HCs and HoCs were nurses (64.0% and 42.6%, respectively). At CUL, Sendwe and the GRHs, midwives represented the second largest category of staff by numbers (32.0% to 34.1%); they were rare in the maternity wards of HoCs and HCs (at most 10.0% of all the obstetric staff). In the HCs, it is nurses (64.0%) and general practitioners (18.6%) who comprise the largest numbers. Obstetricians and other skilled birth attendants represent, respectively, 2.3% and 3.4% of the staff. In the HoCs, surgeons (0.6%) were also present in the maternity wards in addition to obstetricians (0.6%) and general practitioners (15.8%).

### Supplies and equipment for EmONC

Table 1 shows that health facilities in Lubumbashi with a maternity ward had basic supplies and equipment; however, essential supplies and equipment for performing certain EmONC functions were not available in all the surveyed facilities. Only 28.3% had ventouse and forceps, 34.0% had Ambu bags, and 45.3% magnesium sulphate. Overall, the availability of supplies and equipment varied depending on the type of facility. Equipment was more frequently available in the THs, HoCs and GRHs than in the HCs. In more than 30.0% of facilities, there were no sterile platforms. Similarly, there was no transfer forceps in more than 20.0% of health facilities surveyed, with the HCs and HoCs mostly lacking these supplies.

**Table 1:** Availability of supplies and equipment for the management of obstetric and neonatal emergencies, 53 health care facilities, Lubumbashi, 2010

Equipment and/or drugs	All facilities % (*n* = 53)	Type of health facility			
		HCs % (*n* = 33)	HoCs % (*n* = 9)	GRHs % (*n* = 9)	THs %(*n* = 2)
Equipment					
Delivery table	92.5 (49)	90.9 (30)	88.9 (8)	100.0 (9)	100.0 (2)
Surface for the newborn	79.3 (42)	75.8 (25)	66.7 (6)	100.0 (9)	100.0 (2)
Instrument table	73.6 (39)	69.7 (23)	88.9 (8)	66.7 (6)	100.0 (2)
Curettage kit	88.7 (47)	90.9 (30)	77.8 (7)	88.9 (8)	100.0 (2)
Transfer forceps	81.1(43)	75.8 (25)	77.8 (7)	100.0 (9)	100.0 (2)
Baby-weighing scales	88.7 (47)	84.8 (28)	88.9 (8)	100.0 (9)	100.0 (2)
Sterile platform	69.8 (37)	63.6 (21)	77.8 (7)	77.8 (7)	100.0 (2)
Bag valve masks (Ambu)	34.0 (18)	24.2 (8)	44.4 (4)	55.6 (5)	50.0 (1)
Heating surface	22.6 (12)	12.1 (4)	44.4 (4)	22.2 (2)	100.0 (2)
Aspirator	56.6 (30)	48.5 (16)	77.8 (7)	55.6 (5)	100.0(2)
Oxygen source	30.2 (16)	24.2 (8)	33.3 (3)	33.3(3)	100.0 (2)
Incubator	26.4 (14)	9.1 (3)	55.6 (5)	44.4 (4)	100.0 (2)
Phototherapy apparatus	9.4 (5)	0.0 (0)	11.1 (1)	22.2 (2)	100.0 (2)
Fridge for the blood bank	20.5 (11)	9.1 (3)	44.4 (4)	22.2 (2)	100.0 (2)
Ventouse & forceps	28.3 (15)	24.2 (8)	22.2 (2)	44.4 (4)	50.0 (1)
Drugs					
Oxytocin	98.1 (52)	100.0 (33)	88.9 (8)	100.0 (9)	100.0 (2)
Magnesium sulphate	45.3 (24)	30.0 (10)	55.6 (5)	77.8 (7)	100.0 (2)
Injectable antibiotics	96.2 (51)	97.0 (32)	88.9 (8)	100.0 (9)	100.0 (2)
Blood products	45.3 (23)	33.3 (11)	77.8 (7)	44.4 (4)	100.0 (2)
Nasogastric tube	37.7 (20)	27.3 (9)	55.6 (5)	44.4 (4)	100.0 (2)
Electrolytes for infusion	92.5 (49)	90.9 (30)	88.9 (8)	100.0 (9)	100.0 (2)

*HC* health centre, *HoC* hospital centre, *GRH* general referral hospital, *TH* tertiary hospital

### Provision of emergency obstetric and neonatal care

Table 2 shows that 73.6% of 53 facilities had administered parenteral antibiotics, 79.2% had systematically offered oxytocics, 39.6% had administered magnesium sulfate, 73.6% had manually evacuated placentas, 81.1% had removed retained placenta products, 54.7% had revived newborns, 35.8% had performed caesarean sections, and 47.2% had performed blood transfusions. Function 6, vaginal delivery assisted by ventouse or forceps, was the least-performed function overall (3.8% of facilities). It was performed in only two facilities: one HoC and Sendwe Hospital. Administration of magnesium sulphate, neonatal resuscitation, blood transfusion and caesarean section were the next most infrequently performed functions. We observed, nevertheless, that several HCs had performed caesarean sections and blood transfusions and had administered anticonvulsants for women who presented with complications.

**Table 2:** Provision of emergency obstetric and neonatal care, Lubumbashi, 2010

Functions		All facilities %(*n* = 53)	Type of health facility			
			HCs % (*n* = 33)	HoC % (*n* = 9)	GRHs % (*n* = 9)	THs % (*n* = 2)
1	Parenteral administration of antibiotics	73.6 (39)	63.6 (21)	77.8 (7)	88.9 (8)	100.0 (2)
2	Intramuscular administration of uterotonic drugs	79.2 (42)	81.8 (27)	77.8 (7)	66.7 (6)	100.0 (2)
3	Magnesium sulfate administration	39.6 (21)	21.2 (7)	55.6 (5)	77.8 (7)	100.0 (2)
4	Manual removal of the placenta	73.6 (39)	69.7 (23)	88.9 (8)	66.7 (6)	100.0 (2)
5	Removal of retained placenta products	81.1 (43)	75.8 (25)	88.9 (8)	88.9 (8)	100.0 (2)
6	Assisted vaginal delivery using vetouse or forceps	3.8 (2)	0.0 (0)	11.1(1)	0.0 (0)	50.0 (1)
7	Neonatal resuscitation using a bag and mask	54.7 (29)	36.4 (12)	77.8 (7)	88.9 (8)	100.0 (2)
8	Blood transfusion	47.2 (25)	24.2 (8)	77.8 (7)	88.9 (8)	100.0 (2)
9	Caesarean section	35.8 (19)	24.2 (8)	44.4 (4)	55.6 (5)	100.0 (2)

**Fig. 2:**
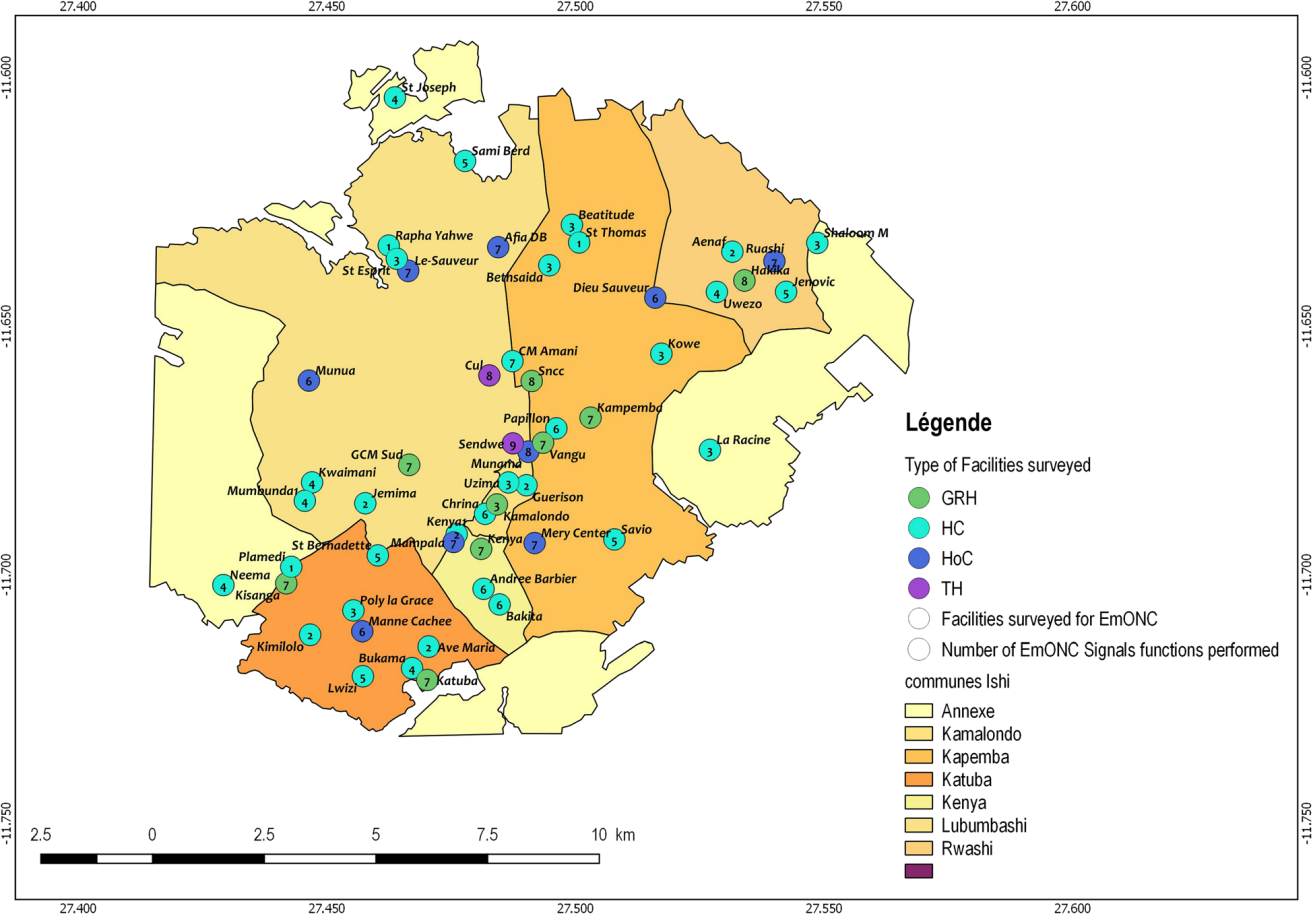
Map of health care facilities by number of EmONC signal functions provided in the 3 months prior to the survey (Our map)

In Fig. 2, we show that none of the HCs performed all the BEmONC functions in the specified period; two thirds (69.7%; 23/33) performed at least four signal functions, 18.2% (6/33) performed only two functions, 9.1% (3/33) offered only one function, while 3.0% (1/33) of facilities performed none of the functions. None of the HCs performed vaginal delivery assisted by ventouse or forceps. As for CEmONC, only Sendwe Hospital performed all nine signal functions during the 3 months preceding the survey. Nearly half (45.0%; 9/20) of facilities supposed to offer CEmONC offered only seven signal functions. Nearly 35.0% (7/20) of the surveyed facilities offered EmONC functions 8–9, in addition to other BEmONC signal functions.

As shown in Table 3, procedures relating to newborn care were not performed frequently during the period prior to the survey, with the exception of antibiotic administration. Feeding newborns with a nasogastric tube, thermal protection of newborns with kangaroo mother care or an incubator, and oxygen therapy and transfusions of newborns were rarely carried out. The various types of health facilities showed differences in which newborn procedures were offered. These procedures were more frequently performed in HoCs and GRHs than in other health facilities. Although several of these procedures were only recommended or indicated to be conducted in referral health facilities (GRH and THs), several HCs were offering them to women and newborns. This was the case for oxygen treatment, thermal care using an incubator, transfusion of newborns and use of nasogastric tubes for feeding. Neonatal intensive care is in the scope of reference facilities, but we observed certain private HC facilities also offered these services when, in principle, they should have referred the cases to a higher level of care.

**Table 3:** Delivery of obstetric and neonatal care not included in the EmONC signal functions, Lubumbashi, 2010

Procedure	All facilities %(*n* = 53)	Type of health facility			
		HCs % (*n* = 33)	HoC % (*n* = 9)	GRHs % (*n* = 9)	THs % (*n* = 2)
AMTSL	50.9 (27)	45.5 (15)	55.6 (5)	55.6 (5)	100.0 (2)
Antibiotic administration	90.6 (48)	93.9 (31)	88.9 (8)	100.0 (9)	0.0 (0)
Nasogastric tube feeding	17.0 (9)	9.1 (3)	22.2 (2)	22.2 (2)	100.0 (2)
Kangaroo mother care	37.7 (20)	39.4 (13)	33.3 (3)	33.3 (3)	50.0 (1)
Thermal protection using an incubator	18.9 (10)	3.0 (1)	44.4 (4)	33.3 (3)	100.0 (2)
Newborn oxygen therapy	24.5 (13)	15.2 (5)	33.3 (3)	33.3 (3)	100.0 (2)
Newborn blood transfusion	22.6 (12)	12.1 (4)	22.2 (2)	44.4 (4)	100.0 (2)
Audits of maternal deaths	13.2 (7)	9.1 (3)	0.0 (0)	33.3 (3)	50.0 (1)
Use of referrals‡	92.2 (47)	97.0 (32)	88.9 (8)	77.8 (7)	0.0 (0)
Means of transportation used for referral					
Ambulance	14.9 (7)	15.6 (5)	12.5 (1)	14.3 (1)	†
Public transport	80.9 (38)	78.1 (25)	87.5 (7)	85.7 (6)	†
Walking, cycling or motor biking	4.2 (2)	6.3 (2)	0.0 (0)	0.0 (0)	†

‡: Referral facility (*n* = 51); *AMTSL* active management of the third stage of labour, *HC* healthcare centre, *GRH* general referral hospital, *TH* Tertiary hospital ; †: not calculated for referral healthcare facilities

With regards to referrals, health facilities made more use of public transport for the referral of complicated cases than they did of ambulances. In public transport, the woman rides in a taxi shared with up to five others or a minibus shared with up to 24 others. In some cases, if the family has the means, they may hire a car in which she is transported accompanied by family members. In either case, she is not accompanied by health care staff and is responsible for the costs of transport. It was also noted that 4.1% of facilities sent patients presenting with complications for referral by foot, bicycle or motorbike. The proportion of referrals carried out by ambulance did not differ between the HCs (15.6%), the GRHs (14.3%) and the HoC (12.5%) (p = 0.91). However, only the HCs used bikes or motorbikes for the referral of complicated cases.

### Reasons for the EmONC functions not being provided

In one case out of four (25.3%), the reason a given EmONC function was not provided during the 3 months preceding the survey was that the function was not indicated at the facility during that time (Additional file 3 gives the number of deliveries as well as the complications experienced during the 3 months preceding the survey). Other reasons included lack of supplies and equipment (26.7%), refusal to change the established routines (26.8%), lack of skilled staff (21.1%) and management issues (1.1%).

### Availability, use and quality of EmONC

Table 4 shows that only one facility (Sendwe Hospital, a TH) provided all CEmONC signal functions in the preceding 3 months. When considering all the facilities in Lubumbashi that have a maternity care unit, we observed from the review of all admissions in 2010 that out of 9294 women admitted, 3820 women presented with DOCs and 366 with indirect obstetric complications (IOC). Among women who underwent a Caesarean section, 30.3% did so at Sendwe. In total, there were 57 maternal deaths due to DOCs and 19 maternal deaths due to IOCs. The number of maternal deaths was higher in facilities that had provided only some of the EmONC functions compared to Sendwe Hospital. No maternal deaths due to IOCs were reported in the Sendwe hospital.

**Table 4:** Use of EmONC and foetal-maternal and neonatal outcomes in relation to facility type, Lubumbashi, 2010

Indicators (number)	All facilities surveyed				Estimates for Lubumbashi^‡^			
	Numbers [1]	CEmONC [2]	BEmONC [3]	Partial [4]	Numbers (95% CI) [5]	CEmONC (95% CI) [6]	BEmONC [7]	Partial (95% CI) [8]
Total population (A)	918,819^†^				1,548,923			
Health facility surveyed (B)	53	1	0	52	180	2	0	178
Expected births (C)^a^	36,753	-		-	61,957	-		-
Total births (D)	32,573	2489		30,084	54,911 (54,534–55,292)	4196 (4167–4225)		50,715 (50,367–51,068)
Live births (E)	32,142	2300		29,842	54,184 (53,632–54,561)	3877 (3851–3904)		50,307 (49,961–50,657)
Expected complications (F)^b^	5513	-		-	9294 (9230–9358)	-		-
Direct obstetric complications (DOC) managed (G)	2266	387		1879	3820 (3794–3847)	652 (648–657)		3168 (3146–3189)
Indirect obstetric complications (IOC) managed (H)	217	38		179	366 (363–368)	64 (63–65)		302 (300–304)
Number of caesarean sections (I)	1109	336		773	1870 (1856–1882)	566 (562–570)		1303 (1294–1312)
Maternal deaths	45	15		30	76 (75–77)	25 (24–26)		51 (50–52)
*Direct (J)*	34	15		19	57 (56–58)	25 (24–26)		32 (31–33)
*Indirect (K)*	11	0		11	19 (18–20)	0		19 (18–20)
Foetal deaths (L)	315	50		265	531 (527–535)	84 (83–85)		447 (444–450)
Neonatal deaths ≥2500g <24 h (M)	129	78		51	217 (215–218)	131 (130–132)		86 (85–87)
Perinatal deaths (N)	1139	495		644	1920 (1907–1933)	834 (829–840)		1086 (1078–1093)
Non-intrapartum deaths (O)	695	367		328	1172 (1163–1180)	619 (614–623)		553 (549–557)

^†^Population covered by the selected facilities; ^a^4% of the total population; ^b^15% of expected births; ^‡^numbers estimated based on an estimation factor (0.5932); *BEmONC* basic care, *CEmONC* comprehensive EmONC, -not calculated

In 2010, of the 748 fetal intrapartum deaths occurring in all health facilities with a maternity unit, 28.7% occurred at Sendwe. There were 1920 perinatal deaths, of which 834 (43.4%) occurred in that facility. The number of non-intrapartum deaths (i.e., antepartum deaths and neonatal deaths between 1 and 7 days) was also higher at Sendwe Hospital (619 of which 58 (9.4%) were in referred newborns) than in facilities which did not provide CEmONC (553). Table 5 describes the EmONC indicators in Lubumbashi in 2010.

**Table 5:** Indicators of availability, use and quality of EmONC, Lubumbashi, 2010

Indicators^‡, ¥^	Surveyed facilities		
	All	CEmONC	Partial
Availability of EmONC for 918,819 inhabitants *(B2/A1)*	-	1	-
Proportion of births/ EmONC facilities *(D2/D1)*	-	7.6	92.4
Met need for EmOC (% ; *G2/F1*)	7.0	-	-
Incidence of caesarean sections (% ; *I1/C1*)	3.0	0.9	-
Direct obstetric case fatality rate (% ; *J1/G1*)	1.5	3.9	1.0
Indirect obstetric case fatality rate (% ; *K1/H1*)^†^	5.1	0.0	6.1
Proportion of maternal deaths due to IOCs (% ; *K1/J1*)	24.4	0.0	36.7
Intrapartum mortality (% ; (*L1 + M1)/D1*)	1.4	5.1	1.1
Non-intrapartum deaths (% ; *O1/N1*)^†^	61.0	74.1	50.9

^‡^Calculated based on the data from table VI; ^¥^The calculated indicators for the surveyed facilities can be validated by changing the index for each type of indicator; -not calculated; *DOC* direct obstetric complication, *IOC* indirect obstetric complication; ^†^not an EmONC indicator

The data generated using our sample set of facilities was extrapolated and extended to the whole of Lubumbashi, where we found that the EmONC indicators remained the same. Thus, Table 5 shows the number of structures that had offered CEmONC − presented in Table 4 − corresponded to a coverage of one facility with CEmONC per 918,819 inhabitants.

With regards to use of EMONC facilities for deliveries, we observed that only 7.6% of births in 2010 took place in the Sendwe hospital; the other 92.4% took place in facilities that offered some, but not all, EmONC signal functions. Similarly, only 7.0% of complications were managed at the Sendwe hospital in 2010. The overall incidence of caesarean sections was 3.0% among women cared for in the surveyed health care facilities. However, the caesarean sections performed at Sendwe represent only 0.9% of all expected births in Lubumbashi in 2010.

Maternal mortality due to DOCs was 3.9% at Sendwe. This was four times higher than that observed in facilities with partial EmONC. Mortality resulting from IOCs was higher than that associated with DOCs. The intrapartum mortality in Sendwe was 5.1%. Similar to what was found for maternal mortality, intrapartum mortality was twice as high in this facility compared to facilities with partial EmONC. This observation is also true for the rate of non-intrapartum deaths, which accounted for over half (61.0%) of perinatal deaths. At Sendwe, the proportion of non-intrapartum deaths was higher (74.1%) than that in facilities providing only partial EmONC (50.9%; *p* < *0.001*).

### Maternal complications and the causes of maternal deaths

**Fig. 3:**
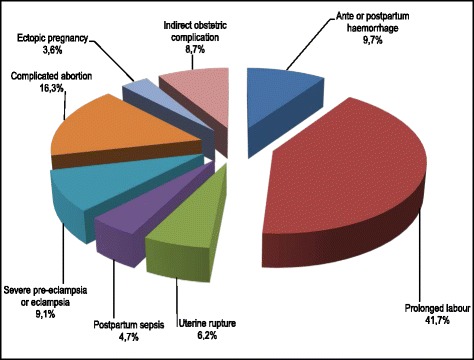
Obstetric complications affecting women in labour, Lubumbashi, 2010

The complications that affected women occurred with varying frequencies. Ranked by their frequency in 2010, they were: prolonged labour (41.7%), complications linked to abortions (16.3%), haemorrhage during labour (9.7%), severe pre-eclampsia or eclampsia (9.1%), uterine rupture (6.2%) and postpartum sepsis (4.7%). IOCs accounted for 8.7% of all complications seen in women attending maternity clinics in Lubumbashi in 2010 (Fig. 3).

**Fig. 4:**
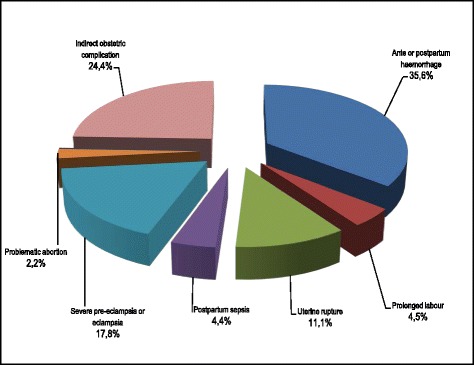
Causes of maternal deaths in Lubumbashi, 2010

**Fig. 5:**
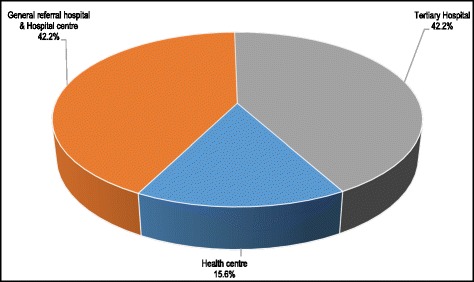
Distribution of maternal deaths by type of health facility, Lubumbashi, 2010

Ranked by their frequency, the main causes of maternal deaths in Lubumbashi in 2010 were ante- and postpartum haemorrhage (35.6%), severe eclampsia or pre-eclampsia (17.8%), uterine rupture (11.1%), postpartum sepsis (4.4%) and complications relating to abortion (2.2%). IOCs accounted for 24.4% of maternal deaths during the same period (Fig. 4). In Fig. 5, the number of maternal deaths occurring in the TH was equivalent to all the deaths in all the GRH (10) and the HoCs (9); only 15.6% of deaths occurred in the HCs.

**Fig. 6:**
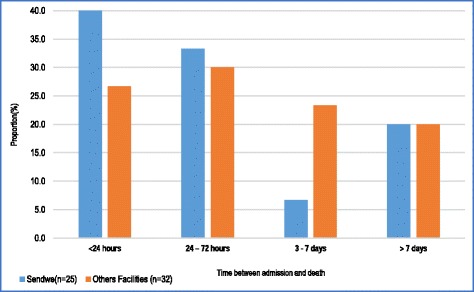
Time elapsed between admission and death of women with direct Obstetric complications in the maternity units of surveyed health care facilities

Figure 6 shows that in the maternity unit at Sendwe, 73.3% of maternal deaths occurred during the 72 h following admission (40% in less than 24 h and 33.3% between 24 and 72 h) and 26.7% between 72 h and more than 1 week. The proportion of maternal deaths occurring in the course of the first 72 h was higher at Sendwe than at other health facilities (73.3% vs 56.7%; p < 0,001).

## Discussion

The EmONC functions are a service package proven to have an impact on the reduction of maternal and perinatal mortality [3, 10–13, 34]. In Lubumbashi, availability of EmONC is limited. This study found one health care facility providing the nine signal functions of comprehensive EmONC for 918,819 inhabitants. Apart from the tertiary hospital (Hospital Sendwe), no other facility provided all seven signal functions of BEmONC. This falls short of WHO’s recommended minimum, which indicates that there should be five EmONC facilities and at least one facility providing CEmONC for a population of this size [10].

What explains this low EmONC distribution despite this being a mostly urban health district [29] is that most health facilities do not perform vacuum extractions, since some providers consider them dangerous. If signal function 6, assisted vaginal delivery using vacuum (ventouse) or forceps extraction, was not taken into account in our assessment of EmONC availability, our results would show five facilities providing CEmONC for 500,000 inhabitants, which meets the WHO recommendation. In this study, we observed that several HCs that were willing to perform vacuum extraction instead carried out Cesarean sections, even though it was not within their scope of practice. The fact that certain health care facilities –particularly private ones– that are considered HCs, that is, the lowest level of service delivery, perform Caesareans poses a problem, on the one hand, in tracking the availability and quality of EmONC functions, and on the other hand, in terms of management of information available in the health care system. In fact, with the disengagement of the state from the financing of health care, the private sector has registered more and more facilities, to the point that more than 60% of health care facilities in Lubumbashi are private [30]. The weak regulation of health care provision and the medicalization of services at primary health care centers has engendered an overlap of technical services, and competition between the primary and secondary and between the secondary and tertiary levels. The results of the present study show that an evaluation of EmONC based on the presumption that HC offer only BEmONC while only GRH offer CEmONC is insufficient to determine exactly which EmONC functions are provided. In the context of a health care system dominated by the private sector and little regulated, as is the case in Lubumbashi [30], in the context of evaluating availability, use, and quality of EmONC, all the EmONC signal functions – especially cesarean, which is often performed at primary-care facilities for the economic survival of personnel – should be systematically researched in private-sector facilities that handle deliveries, regardless of their status in the health care system hierarchy (HC, HoC, or GRH).

This observation implies the reconsideration of the role of primary and secondary facilities vis-à-vis the tertiary GRHs in terms of collaboration, complementarity, and management of information in the HZ. However, in an environment where caesarean section is less available, assisted vaginal delivery becomes all the more important. This is the case in the city of Lubumbashi, where dystocia, certain forms of which can be managed using the ventouse, represents 40% of all delivery complications.

When assessing the availability of supplies and equipment for the management of obstetric complications, we found that 28.3% of the facilities had ventouse devices (Table 1). However, the fact that these were not being used despite there being an indication for it, raises the issue of staff training and of the decisions that the maternity staff make when it comes to the choice of procedures. The reluctance of health care staff to use assisted vaginal delivery for fear of maternal and neonatal complications is unjustified. It reveals a lack of provider training and experience, both of which are determinants of the outcome of vacuum- or forceps-assisted delivery [16, 19]. In Lubumbashi, this lack of training is one of the main obstacles to the introduction of vacuum extraction by ventouse as the tool of choice for instrument-assisted delivery. This is due to the lack of attention given to this technique in training of doctors, midwives, and nurses, both in their schooling and on the job, and suggests that programs to train providers in vacuum extraction by ventouse should be developed in the context of the internship or on the job. Professional organizations should be mobilized to ensure this training, and content should be based in part on hands-on simulation. Adoption of vacuum extraction by ventouse as a first-line intervention should be encouraged only after a minimum standard of training has been attained.

The observation that more health facilities offer signal functions 8–9 (blood transfusions and caesarean sections) than either BEmONC or CEmONC supports the finding that some services are preferred due to their financial returns [35, 36]. Similar observations have been reported by Lawn et al. in West Africa [3]. However, the fact that not using the ventouse or forceps prevents a facility from being considered as offering EmONC services raises the question of the applicability of this function. In the case of certain forms of dystocia, for example, the use of forceps or the ventouse is one alternative. But more often than not, in facilities where it is possible to do a cesarean, staff prefer this over extraction by ventouse. In this case, for the same indication, staff have the choice between the cesarean and the ventouse. This choice automatically affects the provision of EmONC functions insofar as the two will not be used simultaneously. This consideration suggests that if extraction by ventouse, forceps, and cesarean are, in certain circumstances, alternatives, then one might well consider that EmONC is available in Lubumbashi, but one alternative, cesarean section, is preferred over the others.

Thus, staff training and the regulation of how obstetric and neonatal care is provided are both essential features which, if improved, would help ensure that women and newborns obtain health interventions in the most cost-effective way.

Regarding the use of EmONC, we noted that Lubumbashi is dotted with many health facilities, but only a small proportion of facility births (7.6%) take place in health facilities providing CEmONC, while 92.4% take place in facilities that offer some, but not all, EmONC signal functions. In Table 3, more than 90.0% of health facilities claimed to have referred complicated cases to a higher-level facility. While this information might seem to imply that obstetric complications are appropriately managed within the health system, information is still lacking concerning the proportion of cases referred relative to the number of patients admitted to the facility, and concerning the time it takes the staff within the lower-level facility to refer their complicated cases. Very often, the referral is delayed due to false optimism and indecisiveness within these lower-level health facilities, or due to unavailable or inappropriate transportation [20]. Even though the facilities report that they refer, in this context, this cannot be understood to imply all the necessary conditions (transport, accompaniment by a health care provider and a referral note); referral is rather a decision informing the family to go to such and such health facility, sometimes without precise information. Women are often not accompanied and there is often no transport. Given that she is not accompanied by a staff member from the referring facility, the decision to go or not to the indicated facility depends on the woman or her family. It is therefore not guaranteed that the woman who is referred to *x* facility will arrive there. Going there or not depends on her ability to find transport and pay the presumed costs of care, and on her perception of the quality of care in the facility.

As indicated by WHO [10], all women who had obstetric complications delivered in facilities that offered at least one EmONC signal function during the 3 months that preceded our survey. However, this indicator is not very appropriate, since admission to a health facility following a DOC does not necessarily guarantee satisfactory care. The word “satisfactory” here refers to the principle that care is respectful, but also that the time between the arrival of a woman in a facility and her treatment is short. Such timeliness is only possible if skilled staff is present, and if the appropriate supplies and equipment are available. However, since several health facilities in Lubumbashi do not have the necessary emergency kits for the management of complicated cases, and rely on the families of women to obtain these [37], it is clear that the delay is inevitably long. This delay could contribute to increased rates of maternal mortality or intrapartum mortality [10].

The low incidence of Caesarean sections relative to the number of expected births reported in this study is another indicator that EmONC is not frequently used by providers to care for women who present with complications. While this incidence is expected to range from 5 to 15% [10], we found a rate of 3% among the women cared for in the surveyed health care facilities. In the context of Lubumbashi, this low rate of cesareans can be explained in several ways; the most common explanation put forward by women is the high cost [35, 36]. From another point of view, taking into account the proportion of facility births (≈95%) [36] and the medicalization of first-line facilities in Lubumbashi [30], this low rate could be explained by the fact that many women for whom cesareans are indicated delivered in facilities not authorized to provide them, but no information about them (women) was mentioned in the official documents of the health facilities [36, 38].

The quality of care for women who gave birth in the health facility that offered CEmONC was not good. The reported maternal mortality due to DOC (3.9%) was higher than the acceptable level (≤1%). This reflects the lack of supplies, drugs and equipment, as well as the lack of skilled staff. We observed, for example, that out of all health facilities, only the THs possessed the necessary supplies or equipment for the management of obstetric complications. It was indeed in one of these facilities that all of the EmONC functions were offered. We would therefore expect that EmONC would be of a good quality. However, there is a need to distinguish between availability, sufficiency and the quality of supplies and equipment [3]. At Sendwe Hospital, even if the staff are supposedly skilled, given that supplies such as surgical kits, blood, and intravenous fluids must often be sought by the family of the parturient, the time needed to acquire them contributes to prolonging the considerable delay linked to the overwhelm of the first-line facilities (the so-called third delay), to the means of transport used to reach the health facility (second delay), and the decision, by the woman and/or her family, to seek care (first delay) [31, 35].

When we look through the causes of reported maternal deaths (Fig. 3) and the interval between admission and death (Fig. 6), we can see that these delays are generally the basis for fatal outcomes. Indeed, according to Filippi [39], none of these complications would be fatal (in a 12-h timeframe) if they were referred in time, and if the receiving facility was sufficiently equipped and organized to deal with any kind of emergency. Thus, even when the CEmONC functions were provided at Sendwe, we do not know how long after her arrival a woman was cared for.

These considerations are equally valid in explaining intrapartum mortality. We have noted that the number of reported intrapartum deaths and maternal deaths by DOC was higher in the CEmONC facility than in those which provided only some EmONC functions. This difference is explained by the characteristics of referrals to CEmONC facilities. These facilities are often considered a last resort – even for the other GRHs – when complications cannot be managed at the first level of resort. It is thus obvious that the number of deaths would be higher – though still under the acceptable threshold─ in these facilities than in other facilities.

IOCs accounted for only 24.4% of all maternal deaths, but non-intrapartum deaths represented more than half of all perinatal deaths (61.0%) reported in this study. The proportion of non-intrapartum deaths was higher in Sendwe (74.1%) than in facilities that offered only partial EmONC (50.9%) (Table 5). This situation implies that, EmONC alone is not enough; even facilities providing EmONC need to improve the quality of care given to newborns who have problems requiring interventions other than those featured in the EmONC signal functions [3, 38, 40]. Only urgent neonatal complications are considered in the EmONC signal functions, with others not being taken directly into account [10]. For example, although prematurity and low birth weight are the leading causes of neonatal and perinatal death, these cannot be prevented or appropriately managed by the health procedures included in the EmONC functions. The primary aim of the EmONC functions is to manage respiratory distress. Although we know that prematurity can be secondary to complications such as eclampsia, severe pre-eclampsia or placenta previa, neither magnesium sulphate, oxytocin nor caesarean sections can prevent neonatal death associated with this complication. Other procedures not accounted for by the EmONC functions can address neonatal mortality from non-emergency causes. According to Lee et al. [7, 8], thermal protection of the newborn, the use of oxygen therapy and nasogastric tubes for feeding as well as other emergency care associated with the prevention and treatment of severe sepsis of the newborn could prevent over 80.0% of perinatal deaths. In this study, we focused only on EmONC functions; it would be good to take other services into account when assessing the availability, use and quality of neonatal care in general, rather than solely relying on the EmONC signal functions, which focus on neonatal emergency care (resuscitation). Anecdotally, we can report that these other procedures were not always available in the facilities surveyed, despite there being strong indications for their use during the 3 months period covered by our study.

Our observations concerning EmONC availability, use and quality in Lubumbashi are similar to those made in urban or urban–rural centers in several African countries [16–21]. In all these settings, between 2009 and 2015, signal functions 1 and 2 were the most often performed (70–95%) by health facilities. By contrast, removal of retained products, assisted vaginal delivery, cesarean, and transfusion were the least often performed (3–30%) [17]. Pattinson observed in 12 districts in South Africa that although the provision of EmONC signal functions was higher (≥60%) than that reported in Lubumbashi and in other African settings, vaginal delivery assisted by ventouse was also the function that was least offered both by the health centers and the GRHs (≈60%) [17]. In this study the authors also observed that only 48% of GRHs had provided all the signal functions. In surveys carried out in Madagascar, Mali, Ethiopia, Malawi, Zambia, Uganda, and other African countries, as in Lubumbashi, the proportion of facilities that had provided all these functions did not exceed 30% (10–30%); similarly, the proportion of deliveries in EmONC facilities did not exceed this percentage (10–30%) [16–21, 41]. The proportion of obstetrical needs met and the rates of cesarean sections reported in these studies were also weak and variable, ranging, for the obstetrical needs met, from 9.6% in Madagascar [41] to 48% in South Africa [17], and for cesarean sections, from 1.5 to 9.0%, respectively, in the same countries [17, 41]. The rate of cesarean section observed in our study is also similar to those reported by Chu [20] in Lubutu and Masisi in DRC (≈3.0%), Bo in Sierra Léone and in Kabezi in Burundi, where it remains generally lower than the minimum recommended by the WHO [10].

This study, which involved staff interviews and the analysis of archival data, has some limitations. It is possible that members of staff forgot to mention a particular procedure which was carried out during the period of study. This could have changed our classification of the facilities, which was based on whether or not they had provided EmONC functions. It was to limit this recall bias that we chose to record the provision of EmONC functions during a relatively short 3 months window.

The relationship between EmONC signal functions and maternal indications is likely to have affected the proportion of health care facilities that accomplished a specific EmONC function. For example, concerning the reasons that justified the lack of provision of EmONC, 25.3% of the maternity ward managers acknowledged not having had indications to offer it. This remains debatable. First of all, if functions 1 and 3–9 are offered in the context of complications, function 2 is systematically offered preventively to all women delivering. Not having administered uterotonics on the pretext of lack of indication shows a lack of information due to the fact that these providers have not been trained about AMTSL – even if they did not want to say so. This is also the case for facilities that conducted caesareans, but where the administration of antibiotics was not mentioned among the functions offered (Additional files 1 and 3).

Secondly, for certain types of dystocias, cesarean and vacuum-assisted delivery are two management alternatives; however, the cesarean is preferred by providers. Thirdly, most functions specific to certain complications can also be indispensable for others [10]. Blood transfusion is a fundamental function in case of hemorrhage due to placental retention, uterine rupture, complications of abortion, or during a cesarean, the management of extra uterine pregnancy and also the severe anemias observed in poor countries [34], and parenteral antibiotics are similarly offered as infection prevention in case of uterine rupture, after a surgical intervention, complications of abortion, and post-partum hemorrhage after placental retention. This is not the case for magnesium sulfate, which is very specific for eclampsia. These relationships raise the issue of adjustment or not of EmONC functions according to complications.

With regards to the quality of the data collected, only one registry existed within the HCs, and this contained information about the facility’s main activities. In this registry, several columns were added and held information about maternal complications and maternal-foetal outcomes. In other health facilities, complications relating to abortions were sometimes recorded in a general care registry, interspersed with other care procedures. Newborns practically never had their own file, the information instead being written into the mother’s file, which otherwise contained data concerning her stay. The data presented here was extracted from these documents, held by the health facilities. It is therefore possible that we underestimated the use of the EmONC services due to poor recordkeeping. Nevertheless, we attempted to reduce this uncertainty by first liaising with the maternity ward manager so that he or she could confirm the validity of the records, and also by triangulating independent sources of data management tools: i) maternity registers filled out by the maternity ward manager and her colleagues; ii) admission registers that manage the data from the maternity unit using files entered before admission and discharge of the women, on the basis of which the NSIS reports are developed iii) and the prevention of mother-to-child transmission (PMTCT) of HIV registry [3]. In fact, the health care facilities included in this study are among those in which we have integrated PMTCT activities since 2004. An HIV screening registry of parturients was maintained independently of the maternity register by the facility’s point person for this activity. Given the high quality of these records, this was considered a reference to verify the completeness of the data.

All the reference facilities were included in the study, so the extrapolation applies only to health centers and hospital centers. Given that the extrapolation was performed on the basis of the data from surveyed facilities, it is possible that it doesn’t reflect the situation of HCs and hospital centers that were not surveyed if their characteristics – urban or urban–rural location and private or public sector – were different from those of surveyed facilities. However, to reduce the effect of this bias, we selected facilities while ensuring that the sample was proportional to their location.

## Conclusion

EmONC availability falls short of WHO standards in Lubumbashi. In this study, we found one comprehensive EmONC facility for 918,819 inhabitants. Apart from the tertiary hospital (Sendwe), no other facility provided all the BEmONC signal functions. All the health care facilities had provided at least one of nine EmONC signal functions during this period. Function 6, assisted vaginal delivery, was the least-performed function overall (3.8% of facilities).

In 2010, all women in the surveyed facilities who had obstetric complications delivered in health care facilities that provided at least one EmONC signal function in the 3 months preceding our survey; 7.0% of these women delivered in the facility which provided CEmONC. In the health facility that provided CEmONC, maternal mortality by DOC was 3.9%, well above the acceptable threshold; intrapartum mortality was also high in this facility (5.1%).

To further reduce maternal and neonatal mortality, and to improve maternal, neonatal and child health, it is important to develop staff skills regarding EmONC, increase the availability of supplies and equipment, and standardise the care processes in all the health facilities in Lubumbashi. The monitoring and assessment of the quality of care given to newborns must go beyond a focus on emergencies to incorporate other indicators of quality of care given to all newborns, such as those with low birth weight, to reduce non-intrapartum deaths. The patient transport system must be strengthened to reduce delays during the referral process of complicated cases. Auditing of maternal and neonatal deaths, as well as near misses, should be established and used as a basis for monitoring the quality of care provided to mothers and newborns.

## Additional files

Additional file 1: Statistics on deliveries and maternal complications in 2010 in the surveyed health care facilities of Lubumbashi. (XLSX 23 kb)
Additional file 2: Data collection tool. (ZIP 106 kb)
Additional file 3: Statistics on deliveries, maternal complications and EmONC signal functions between January and March 2011 in the surveyed health care facilities of Lubumbashi. (XLSX 19 kb)

## Abbreviations

AIDS: Acquired immunodeficiency syndrome
AMSTL: Active management of the third stage of labour
BEmONC: Basic Emergency obstetric and neonatal care
CEmONC: Comprehensive Emergency obstetric and neonatal care
CUL: Cliniques Universitaires de Lubumbashi
DOC: Direct obstetric complications
DRC: Democratic Republic of the Congo
EmONC: Emergency obstetric and neonatal care
GRH: General referral hospital
HC: Health Centre
HIV: Human immunodeficiency virus
HoC: Hospital centre
HZ: Health Zone
IOC: Indirect obstetric complications
MCH: Maternal and child health
MMR: Maternal mortality ratio
NPRH: National Program for Reproductive Health
NSIS: National sanitary Information System
PMTCT: Prevention of mother-to-child transmission
SNCC: Société Nationale Congolaise de Chemins de Fer
TH: Tertiary hospital
WHO: World Health Organization
