# Supplementary material for: Emergency obstetric and neonatal care availability, use, and quality: a cross-sectional study in the city of Lubumbashi, Democratic Republic of the Congo, 2011
Source: BMC Pregnancy Childbirth. 2017 Jan 19;17:40. doi: 10.1186/s12884-017-1224-9 (PMC5244553; doi:10.1186/s12884-017-1224-9)
Supplement: Additional file 2: — Data collection tool. (ZIP 106 kb) [file 12884_2017_1224_MOESM2_ESM.zip › Additionnal file-2-EnglishR4.docx]

**Evaluation of the availability, use and quality of emergency obstetric and neonatal care (EmONC) in the Lubumbashi Health District**

**Date of survey /………………………/ Province /………………………………………../**

**Health District /………………………/ Health Zone /………………………………............./**

**Health Area /………………………/ Name of health facility /……………………............................/**

**Sheet number /………………………/ Name of investigator /………………………………............./**

**Total population of the health zone /…………………………………………………………./**

**Number of health areas in the HZ /…………………………………………………………./**

**Total population of the health area /…………………………………………………………../**

**I. IDENTIFICATION OF THE HEALTH FACILITY**

| ***[Q1]*** | ***[Q2]*** | ***[Q3]*** | ***[Q4]*** | ***[Q5]*** | ***[Q6]*** | ***[Q7]*** |
| --- | --- | --- | --- | --- | --- | --- |
| Code of the head of the healthcare facility (HF) | Gender of the head of the healthcare facility | Occupation of the head of the healthcare facility | Seniority of the head of the healthcare facility (year) | Code of the head of Maternity ward | Sex of the head of Maternity ward | Age of the head of Maternity ward |
| /…………………./ | /…………………./ | /………………../ | /………………../ | /…………………/ | /………………/ | /……………/ |

| ***[Q8]*** | ***[Q9]*** | ***[Q10]*** | ***[Q11]*** | ***[Q12]*** | ***[Q13]*** | ***[Q14]*** |
| --- | --- | --- | --- | --- | --- | --- |
| Basic training of the head of Maternity ward | Seniority of the head of Maternity ward (year) | Have there been deliveries in the healthcare facility during the year 2010  *Yes=1*  *No=2* | **If yes,**  **Continue with the following questions**  **If not, stop immediately.** | In which setting is the healthcare facility?  *Urban = 1*  *Rural = 2*  *Urbano-rural = 3* | Type of healthcare facility:  *General Referral Hospital = 1*  *Referral Health Center = 2*  *Health Center = 3*  *Private clinic = 4*  *Hospital Center = 5*  *Private polyclinic = 6*  *Single Maternity = 7* | Types of organizations operating:  *State = 1*  *Private = 2*  *Private Company = 3*  *Public / company = 4*  *NGO = 5*  *Confessionnal = 6*  *Others = 7* |
| /……………/ | /…………./ | /……………/ | /………………/ | /……………./ | /……………………../ | /……………………./ |

**II. INFORMATION ON HUMAN RESOURCES**

**1. Knowledge about the management of obstetric and neonatal emergencies**

| ***[Q15]*** | ***[Q16]*** | |
| --- | --- | --- |
| During your employment, have you been trained in reproductive health?  *Yes = 1*  *No = 2* | If so, what modules or topics on which you were trained?  *Yes = 1*  *No = 2* | |
| /………./ | Utilization of the partogram | /………./ |
|  | Active management of the 3rd phase of delivery | /………/ |
|  | Management of Antepartum and post-partum haemorrhage | /………/ |
|  | Bood Transfusion safety | /………./ |
|  | Management of severe preeclampsia and eclampsia | /………/ |
|  | Assisted vaginal delivery using ventouse or forceps | /………/ |
|  | Essential Care for Newborn | /………./ |
|  | Management of newborn asphyxia | /………/ |
|  | Management of prematurity (Kangaroo maternal care) | /………/ |
|  | Management of newborn infection | /………./ |
|  | Audit of maternal deaths | /………/ |
|  | Management of drugs stock | /………./ |
|  | Hospital hygiene | /………/ |
|  | Artificial removal of the placenta | /………./ |
|  | Not remember anything | /………/ |

|  | **[Q17]** | **[Q18]** | **[Q19]. How many have been trained on the following topics:** | | | | | | | | | | | **[Q20]** |
| --- | --- | --- | --- | --- | --- | --- | --- | --- | --- | --- | --- | --- | --- | --- |
|  | How much does your healthcare facility use the following agent categories? | Number of agents in obstetrics | Utilization of partogram | Active management of the 3rd phase of childbirth | Management of antepartum and postpartum hemorrhage | Blood Transfusion safety | Assisted vaginal delivery using ventouse or forceps | Essential Care of the Newborn | Management of newborn asphyxia | Management of prematurity (Kangaroo maternal care) | Management of newborn infection | Audit of maternal deaths | Management of drugs stock | How many trained people have left the obstetric service in the past 12 months? |
| Obstetricians | /…………/ | /…………/ | /…………/ | /………/ | /…………/ | /………/ | /…………/ | /…………/ | /…………/ | /…………/ | /………/ | /………/ | /……/ | /…………/ |
| Surgeons | /…………/ | /…………/ | /…………/ | /………/ | /…………/ | /………/ | /…………/ | /…………/ | /…………/ | /…………/ | /………/ | /………/ | /……/ | /…………/ |
| General practitioner | /…………/ | /…………/ | /…………/ | /………/ | /…………/ | /………/ | /…………/ | /…………/ | /…………/ | /…………/ | /………/ | /………/ | /……/ | /…………/ |
| Nurses | /…………/ | /…………/ | /…………/ | /………/ | /…………/ | /………/ | /…………/ | /…………/ | /…………/ | /…………/ | /………/ | /………/ | /……/ | /…………/ |
| Midwives | /…………/ | /…………/ | /…………/ | /………/ | /…………/ | /………/ | /…………/ | /…………/ | /…………/ | /…………/ | /………/ | /………/ | /……/ | /…………/ |
| Lab-technician | /…………/ | /…………/ | /…………/ | /………/ | /…………/ | /………/ | /…………/ | /…………/ | /…………/ | /…………/ | /………/ | /………/ | /……/ | /…………/ |
| Pharmacy Assistant | /…………/ | /…………/ | /…………/ | /………/ | /…………/ | /………/ | /…………/ | /…………/ | /…………/ | /…………/ | /………/ | /………/ | /……/ | /…………/ |
| Other | /…………/ | /…………/ | /…………/ | /………/ | /…………/ | /………/ | /…………/ | /…………/ | /…………/ | /…………/ | /………/ | /………/ | /……/ | /…………/ |

**2. Management of obstetric and neonatal emergencies during the last 3 months (before survey)**

| Which of these activities did midwives perform during the last 3 months (previous survey) | | | *Yes=1*  *No=2* | | If yes, ask to see 5 partographs of the previous month and mention the number of those that are filled. | If not, why this activity has not been achieved?  *Lack of training = 1*  *Problem of materials and equipment = 2*  *Management problem = 3*  *Health policy problem = 4*  *No indication = 5* |
| --- | --- | --- | --- | --- | --- | --- |
| **[Q21]** | Utilization of the partogram | | /……/ | | /……………………………./ | /……………………………………../ |
| **[Q22]** | Parenteral administration of antibiotics | | /……/ | |  | /……………………………………../ |
| **[Q23]** | Displays of indications for parenteral antibiotics (currently) | | /……/ | |  | /……………………………………../ |
| **[Q24]** | Systemic administration of oxytocic after expulsion of the fetus | | /……/ | |  | /……………………………………../ |
| ***[Q25]*** | Administration of uterotonic drugs (ergometrine, methergine, misoprostol) | | /……/ | |  | /……………………………………../ |
| **[Q26]** | Controlled cord traction | | /……/ | |  | /……………………………………../ |
| **[Q27]** | Uterine massage after expulsion of the placenta | | /……/ | |  | /……………………………………../ |
| **[Q28]** | Magnesium sulfate administration to treat pre-eclampsia or eclampsia | | /……/ | |  | /……………………………………../ |
| **[Q29]** | Administration of other anticonvulsants (diazepam) to treat pre-eclampsia or eclampsia | | /……/ | |  | /……………………………………../ |
| **[Q30]** | Manual removal of the placenta | | /……/ | |  | /……………………………………../ |
| **[Q31]** | Removal of retained placenta products (manual aspiration, dilation and curettage) | | /……/ | |  | /……………………………………../ |
| **[Q32]** | Assisted vaginal delivery using ventouse or forceps | | /……/ | |  | /……………………………………../ |
| **[Q33]** | Blood transfusion | | /……/ | |  | /……………………………………../ |
| **[Q34]** | Caesarean-section | | /……/ | |  | /……………………………………../ |
| **[Q35]** | Management of other obstetric emergencies | | /……/ | |  | /……………………………………../ |
| **[Q36]** | Administration of oral antibiotics to newborns | /……/ | |  | | /……………………………………../ |
| **[Q37]** | Feeding preterm newborn using nasogastric tube | /……/ | |  | | /……………………………………../ |
| **[Q38]** | Kangaroo maternal care to low weight newborns | /……/ | |  | | /……………………………………../ |
| **[Q39]** | Thermal care for the newborn using the incubator | /……/ | |  | | /……………………………………../ |
| **[Q40]** | Newborn oxygen therapy | /……/ | |  | | /……………………………………../ |
| **[Q41]** | Neonatal resuscitation using a bag and mask | /……/ | |  | | /……………………………………../ |
| **[Q42]** | Newborn blood Transfusion | /……/ | |  | | /……………………………………../ |
| **[Q42]** | Audit of maternal deaths |  | |  | |  |

**3. Referral of obstetric complications and permanence of services**

| **[Q43]** | **[Q44]** | **[Q45]** | **[Q46]** | **[Q47]** | **[Q48]** | |
| --- | --- | --- | --- | --- | --- | --- |
| In the past 3 months, have you referred women with obstetric complications  *Yes = 1*  *No = 2* | To which referral healthcare facility does it refer (name of structure) | How far is the nearest referral healthcare facility that provides surgical care? | Are the references of the nearest referral healthcare facility displayed?  *Yes = 1*  *No = 2* | What are the most common means of transport for referred patient?  *Ambulance = 1*  *Used taxi = 2*  *Feet / bike / motorbike = 3* | Is there a rollover for the following services:  *Yes = 1*  *No = 2* | |
| /………../ | /……………/ | /……………../ | /……………../ | /………….../ | Maternity | /………/ |
|  |  |  |  |  | Pharmacy | /………/ |
|  |  |  |  |  | Blood Bank | /………/ |
|  |  |  |  |  | Operating room | /………/ |
|  |  |  |  |  | Intensive care unit | /………/ |
|  |  |  |  |  | Anesthesia | /………/ |

**III. MATERIEL ET EQUIPEMENT**

|  | **Materials and Equipment** | **Number** | **Available** | **Good condition** | **Bad condition** |
| --- | --- | --- | --- | --- | --- |
| **[Q49]** | **Delivery table** | /………./ | /………./ | /………./ | /………./ |
| **[Q50]** | **Surface for the newborn** | /………/ | /………/ | /………/ | /………/ |
| **[Q51]** | Instrument table | /………/ | /………/ | /………/ | /………/ |
| **[Q52]** | **Curettage kit** | /………./ | /………./ | /………./ | /………./ |
| **[Q53]** | Delivery Equipment | /………/ | /………/ | /………/ | /………/ |
| **[Q54]** | **Transfer forceps** | /………/ | /………/ | /………/ | /………/ |
| **[Q55]** | **Baby-weighing scales** | /………./ | /………./ | /………./ | /………./ |
| **[Q56]** | **Sterile platform** | /………/ | /………/ | /………/ | /………/ |
| **[Q57]** | **Bag valve masks (Ambu)** | /………/ | /………/ | /………/ | /………/ |
| **[Q58]** | Heating table | /………./ | /………./ | /………./ | /………./ |
| **[Q59]** | **Aspirator** | /………/ | /………/ | /………/ | /………/ |
| **[Q60]** | Oxygen source | /………/ | /………/ | /………/ | /………/ |
| **[Q61]** | Incubator | /………./ | /………./ | /………./ | /………./ |
| **[Q62]** | **Phototherapy apparatus** | /………/ | /………/ | /………/ | /………/ |
| **[Q63]** | **Fridge for the blood bank** | /………/ | /………/ | /………/ | /………/ |
| **[Q64]** | **Ventouse & forceps** | /………/ | /………/ | /………/ | /………/ |
| **[Q65]** | Sphygmomanometer for the delivery room | /………/ | /………/ | /………/ | /………/ |
| **[Q66]** | Fetoscope | /………./ | /………./ | /………./ | /………./ |
| **[Q67]** | Light source suitable for gynecological examination | /………/ | /………/ | /………/ | /………/ |
| **[Q68]** | Blood bag with transfusion kit | /………/ | /………/ | /………/ | /………/ |
| **[Q69]** | Sample collection table | /………/ | /………/ | /………/ | /………/ |

**IV. DRUGS AND REAGENTS**

|  | **Drugs** | **Available in stock** | |
| --- | --- | --- | --- |
|  |  | **Yes** | **No** |
| **[Q70]** | **Oxytocin** | /………./ | /………./ |
| **[Q71]** | Magnesium sulfate | /………/ | /………/ |
| **[Q72]** | Injectable antibiotics | /………/ | /………/ |
| **[Q73]** | Blood products | /………./ | /………./ |
| **[Q74]** | The four markers of transfusion safety | /………/ | /………/ |
| **[Q75]** | Catheter 8F, 10F, 12F | /………/ | /………/ |
| **[Q76]** | Nasogastric tube | /………./ | /………./ |
| **[Q77]** | Epicranian | /………/ | /………/ |
| **[Q78]** | Syringe (1ml, 2 ml, 5 ml, 10ml) | /………/ | /………/ |
| **[Q79]** | **Electrolytes for infusion** | /………./ | /………./ |

**V. MANAGEMENT TOOLS**

|  |  | **Utilization**  *Yes = 1;*  *No = 2* | **Available in stock in service** | |
| --- | --- | --- | --- | --- |
| **[Q80]** | **Information Management Tools** |  | **Yes** | **No** |
| **A** | Antenatal care Register | /………/ | /………/ | /………/ |
| **B** | Partogram | /………/ | /………/ | /………/ |
| **C** | Maternity Register | /………/ | /………/ | /………/ |
| **D** | Register of caesareans | /………/ | /………/ | /………/ |
| **E** | Datasheet EmONC | /………./ | /………./ | /………./ |
| **F** | Flowchart EmONC | /………/ | /………/ | /………/ |
| **G** | Newborn Fact Sheets | /………/ | /………/ | /………/ |
| **H** | Register of sick newborns | /………/ | /………/ | /………/ |
| **I** | Sheet for audit of maternal deaths | /………/ | /………/ | /………/ |
| **J** | Sheet for audit of neonatal deaths | /………/ | /………/ | /………/ |
| **K** | Reference ticket | /………/ | /………/ | /………/ |
| **L** | Postnatal Register | /………/ | /………/ | /………/ |
| **M** | Hemovigilance Sheet | /………/ | /………/ | /………/ |
| **N** | Blood Products Request Form | /………/ | /………/ | /………/ |
| **O** | Inventory Sheets for Blood Products | /………/ | /………/ | /………/ |

**2. Quality of register data**

| **[Q81]** | **Register of** | **Are all columns in the register filled?** | | | **Is the data up to date?** | | |
| --- | --- | --- | --- | --- | --- | --- | --- |
|  |  | **Yes** | **No** | **Information not available** | **Yes** | **No** | **Information not available** |
| **A** | Delivery room | 1 | 0 | 9 | 1 | 0 | 9 |
| **B** | Abortions | 1 | 0 | 9 | 1 | 0 | 9 |
| **C** | Operating room | 1 | 0 | 9 | 1 | 0 | 9 |
| **D** | Maternity Ward | 1 | 0 | 9 | 1 | 0 | 9 |
| **F** | Caesarean-section | 1 | 0 | 9 | 1 | 0 | 9 |
| **G** | Sick newborns | 1 | 0 | 9 | 1 | 0 | 9 |
| **h** | Postnatal care | 1 | 0 | 9 | 1 | 0 | 9 |

**VI. USE OF SERVICES**

**1. Information on obstetric and neonatal emergencies in 20...**

| **[Q81]. Indicateurs (cases)** | **January** | **Feb.** | **March** | **April** | **may** | **June** | **July** | **Aug.** | **Septemb.** | **October** | **Novem.** | **Decem.** | **Total** |
| --- | --- | --- | --- | --- | --- | --- | --- | --- | --- | --- | --- | --- | --- |
| New cases at Antenatal care (ANC1) | /………/ | /………/ | /………/ | /………/ | /………/ | /………/ | /………/ | /……/ | /…………/ | /………/ | /………/ | /……… | /…………/ |
| Number of women at ANC4 | /………/ | /………/ | /………/ | /………/ | /………/ | /………/ | /………/ | /……/ | /…………/ | /………/ | /………/ | /……… | /…………/ |
| Total parturients | /………/ | /………/ | /………/ | /………/ | /………/ | /………/ | /………/ | /……/ | /…………/ | /………/ | /………/ | /……… | /…………/ |
| Total live births | /………/ | /………/ | /………/ | /………/ | /………/ | /………/ | /………/ | /……/ | /…………/ | /………/ | /………/ | /………/ | /…………/ |
| Total Caesarean-sections | /………/ | /………/ | /………/ | /………/ | /………/ | /………/ | /………/ | /……/ | /…………/ | /………/ | /………/ | /………/ | /…………/ |
| Total newborns transfused (blood) | /………/ | /………/ | /………/ | /………/ | /………/ | /………/ | /………/ | /……/ | /…………/ | /………/ | /………/ | /……… | /…………/ |
| Pregnant women transfused according to the protocol | /………/ | /………/ | /………/ | /………/ | /………/ | /………/ | /………/ | /……/ | /…………/ | /………/ | /………/ | /……… | /…………/ |
| Parturients transfused according to the protocol | /………/ | /………/ | /………/ | /………/ | /………/ | /………/ | /………/ | /……/ | /…………/ | /………/ | /………/ | /……… | /…………/ |
| **[Q82]. Obstetric complications (cases)** |  |  |  |  |  |  |  |  |  |  |  |  |  |
| Antepartum and post-partum haemorrhage (number) | /………/ | /………/ | /………/ | /………/ | /………/ | /………/ | /………/ | /……/ | /…………/ | /………/ | /………/ | /……… | /…………/ |
| Obstructed labor (number) | /………/ | /………/ | /………/ | /………/ | /………/ | /………/ | /………/ | /……/ | /…………/ | /………/ | /………/ | /……… | /…………/ |
| Uterine rupture (number) | /………/ | /………/ | /………/ | /………/ | /………/ | /………/ | /………/ | /……/ | /…………/ | /………/ | /………/ | /……… | /…………/ |
| Postpartum infection (number) | /………/ | /………/ | /………/ | /………/ | /………/ | /………/ | /………/ | /……/ | /…………/ | /………/ | /………/ | /……… | /…………/ |
| Severe pre-eclampsia or eclampsia(number) | /………/ | /………/ | /………/ | /………/ | /………/ | /………/ | /………/ | /……/ | /…………/ | /………/ | /………/ | /……… | /…………/ |
| Complications related abortion (hemorrhage or infection) | /………/ | /………/ | /………/ | /………/ | /………/ | /………/ | /………/ | /……/ | /…………/ | /………/ | /………/ | /……… | /…………/ |
| Ectopic pregnancy (number) | /………/ | /………/ | /………/ | /………/ | /………/ | /………/ | /………/ | /……/ | /…………/ | /………/ | /………/ | /……… | /…………/ |
| Indirect obstetric complications (number) | /………/ | /………/ | /………/ | /………/ | /………/ | /………/ | /………/ | /……/ | /…………/ | /………/ | /………/ | /………/ | /…………/ |

|  | **January** | **February** | **March** | **April** | **may** | **June** | **July** | **August** | **Septemb.** | **October** | **Novem.** | **Decem.** | **Total** |
| --- | --- | --- | --- | --- | --- | --- | --- | --- | --- | --- | --- | --- | --- |
| **[Q83]. Maternal deaths** | /………/ | /………/ | /………/ | /………/ | /………/ | /………/ | /………/ | /……/ | /…………/ | /………/ | /………/ | /……… | /…………/ |
| **[Q84]. Causes of Maternal Death** |  |  |  |  |  |  |  |  |  |  |  |  |  |
| Antepartum and post-partum haemorrhage (number) | /………/ | /………/ | /………/ | /………/ | /………/ | /………/ | /………/ | /……/ | /…………/ | /………/ | /………/ | /……… | /…………/ |
| Obstructed labor (number) | /………/ | /………/ | /………/ | /………/ | /………/ | /………/ | /………/ | /……/ | /…………/ | /………/ | /………/ | /……… | /…………/ |
| Uterine rupture (number) | /………/ | /………/ | /………/ | /………/ | /………/ | /………/ | /………/ | /……/ | /…………/ | /………/ | /………/ | /……… | /…………/ |
| Postpartum infection (number) | /………/ | /………/ | /………/ | /………/ | /………/ | /………/ | /………/ | /……/ | /…………/ | /………/ | /………/ | /……… | /…………/ |
| Severe pre-eclampsia or eclampsia(number) | /………/ | /………/ | /………/ | /………/ | /………/ | /………/ | /………/ | /……/ | /…………/ | /………/ | /………/ | /……… | /…………/ |
| Complications related abortion (hemorrhage or infection) | /………/ | /………/ | /………/ | /………/ | /………/ | /………/ | /………/ | /……/ | /…………/ | /………/ | /………/ | /……… | /…………/ |
| Ectopic pregnancy (number) | /………/ | /………/ | /………/ | /………/ | /………/ | /………/ | /………/ | /……/ | /…………/ | /………/ | /………/ | /……… | /…………/ |
| Indirect obstetric complications (number) | /………/ | /………/ | /………/ | /………/ | /………/ | /………/ | /………/ | /……/ | /…………/ | /………/ | /………/ | /……… | /…………/ |
| **[Q85]. Fetal and neonatal deaths** |  |  |  |  |  |  |  |  |  |  |  |  |  |
| Neonatal asphyxia (number) | /………/ | /………/ | /………/ | /………/ | /………/ | /………/ | /………/ | /……/ | /…………/ | /………/ | /………/ | /……… | /…………/ |
| Prematurity (low birth weight <2500g) | /………/ | /………/ | /………/ | /………/ | /………/ | /………/ | /………/ | /……/ | /…………/ | /………/ | /………/ | /……… | /…………/ |
| Neonatal sepsis (number) | /………/ | /………/ | /………/ | /………/ | /………/ | /………/ | /………/ | /……/ | /…………/ | /………/ | /………/ | /……… | /…………/ |
| Congenital malformation (number) | /………/ | /………/ | /………/ | /………/ | /………/ | /………/ | /………/ | /……/ | /…………/ | /………/ | /………/ | /……… | /…………/ |
| Number of stillbirths (number) | /………/ | /………/ | /………/ | /………/ | /………/ | /………/ | /………/ | /……/ | /…………/ | /………/ | /………/ | /……… | /…………/ |
| *Antepartum fetal deaths* | /………/ | /………/ | /………/ | /………/ | /………/ | /………/ | /………/ | /……/ | /…………/ | /………/ | /………/ | /……… | /…………/ |
| *Intrapartum fetal deaths* | /………/ | /………/ | /………/ | /………/ | /………/ | /………/ | /………/ | /……/ | /…………/ | /………/ | /………/ | /……… | /…………/ |
| Number of neonatal deaths >2500g (number) | /………/ | /………/ | /………/ | /………/ | /………/ | /………/ | /………/ | /……/ | /…………/ | /………/ | /………/ | /……… | /………/ |
| *<24 hours* | /………/ | /………/ | /………/ | /………/ | /………/ | /………/ | /………/ | /……/ | /…………/ | /………/ | /………/ | /……… | /………/ |
| *≥24 hours* | /………/ | /………/ | /………/ | /………/ | /………/ | /………/ | /………/ | /……/ | /…………/ | /………/ | /………/ | /……… | /………/ |
| Number of neonatal deaths ≤2500g (number) | /………/ | /………/ | /………/ | /………/ | /………/ | /………/ | /………/ | /……/ | /…………/ | /………/ | /………/ | /……… | /………/ |
