# Supplementary material for: Emergency obstetric and neonatal care availability, use, and quality: a cross-sectional study in the city of Lubumbashi, Democratic Republic of the Congo, 2011
Source: BMC Pregnancy Childbirth. 2017 Jan 19;17:40. doi: 10.1186/s12884-017-1224-9 (PMC5244553; doi:10.1186/s12884-017-1224-9)
Supplement: Additional file 2: — Data collection tool. (ZIP 106 kb) [file 12884_2017_1224_MOESM2_ESM.zip › Additionnal file-2-FrenchR4.docx]

## Canevas d’évaluation la disponibilité, l’utilisation et la qualité des soins obstétricaux et néonatals d’urgence (SONU) dans le district sanitaire de Lubumbashi

**Date de l’enquête /………………………/ Province de /………………………………………../**

**District sanitaire de /………………………/ Zone de santé de /………………………………............./**

**Aire de santé de /………………………/ Nom de la structure /……………………............................/**

**Numéro fiche /………………………/ Nom de l’enquêteur /………………………………............./**

**Population totale de la zone de santé /…………………………………………………………./**

**Nombre d’aires de santé de la ZS /…………………………………………………………./**

**Population totale de l’aire de santé /…………………………………………………………../**

**I. IDENTIFICATION DE LA STRUCTURE**

| ***[Q1]*** | ***[Q2]*** | ***[Q3]*** | ***[Q4]*** | ***[Q5]*** | ***[Q6]*** | ***[Q7]*** |
| --- | --- | --- | --- | --- | --- | --- |
| Code responsable de la structure | Sexe du responsable de la structure | Profession du responsable de la structure | Ancienneté du responsable de la structure *(année)* | Code responsable du service de maternité | Sexe du responsable de la maternité | Age du responsable de la maternité |
| /…………………./ | /…………………./ | /………………../ | /………………../ | /…………………/ | /………………/ | /……………/ |

| ***[Q8]*** | ***[Q9]*** | ***[Q10]*** | ***[Q11]*** | ***[Q12]*** | ***[Q13]*** | ***[Q14]*** |
| --- | --- | --- | --- | --- | --- | --- |
| Profession du responsable de la maternité | Ancienneté dans le service *(année)* | Y-a-t-il eu des accouchements dans la FOSA au cours de l’année 2011  *Oui=1*  *Non=2* | **Si oui,**  poursuivez les questions suivantes  **Si non**, arrêtez immédiatement. | Dans quel milieu se trouve la structure ?  *Urbain=1*  *Rural=2*  *Urbano-rural=3* | Type d’établissement :  *Hôpital général de référence=1*  *Centre de santé de référence=2*  *Centre de santé=3*  *Clinique privée=4*  *Centre hospitalier=5*  *Polyclinique privée=6*  *Maternité simple=7* | Types d’organismes exploitant :  *Etat=1*  *Privé=2*  *Entreprise=3 publique/société=4*  *ONG=5*  *Asbl Confessionnel=6*  *Autres=7* |
| /…………………/ | /…………./ | /…………………/ | /………………/ | /……………./ | /……………………../ | /……………………./ |

**II. INFORMATION SUR LES RESSOURCES HUMAINES**

**1. Connaissance sur la prise en charge des urgences obstétricales et néonatales**

| ***[Q15]*** | ***[Q16]*** | |
| --- | --- | --- |
| Avez-vous déjà reçu une formation sur la santé de la reproduction **(personne interrogée)**  *Oui=1*  *Non=2* | Si oui, quels sont à votre mémoire, les modules ou les thèmes qui y sont abordés.  *Oui=1*  *Non=2* | |
| /………./ | Utilisation du partogramme | /………./ |
|  | Gestion active de la 3^ème^ phase de l’accouchement (ocytocine en IM) | /………/ |
|  | Prise en charge de l’Hémorragie ante et post-partum | /………/ |
|  | Sécurité transfusionnelle | /………./ |
|  | Prise en charge de l’éclampsie | /………/ |
|  | Extraction instrumentale par ventouse ou forceps | /………/ |
|  | Soins essentiels du nouveau-né | /………./ |
|  | Prise en charge de l’asphyxie du nouveau-né | /………/ |
|  | Prise en charge du prématuré (soins Kangourou) | /………/ |
|  | Prise en charge de l’infection majeure du nouveau-né | /………./ |
|  | Audit des décès maternels | /………/ |
|  | Gestion des médicaments | /………./ |
|  | Hygiène hospitalière | /………/ |
|  | Délivrance artificielle | /………./ |
|  | Ne se rappelle de rien | /………/ |

|  | **[Q17]** | **[Q18]** | **[Q19]. Combien ont-ils été formés sur les aspects ci-après :** | | | | | | | | | | | **[Q20]** |
| --- | --- | --- | --- | --- | --- | --- | --- | --- | --- | --- | --- | --- | --- | --- |
|  | Combien votre structure utilise-t- elle les catégories d’agents ci-après ? | Nombre d’agents en obstétrique | Utilisation du partogramme | Gestion active de la 3^ème^ phase de l’accouchement | Prise en charge des hémorragies ante et post partum | Sécurité transfusionnelle | Extraction instrumentale (ventouse, forceps) | Soins essentiels du nouveau-né) | Prise en charge de l’asphyxie du nouveau-né | Prise en charge du prématuré (Kangourou) | Prise en charge des infections majeures du nouveau-né | Audit des décès maternels | Gestion des médicaments | Combien de personnes formées ont-elles quitté le service d’obstétrique au cours de 12 derniers mois ? |
| Obstétriciens | /…………/ | /…………/ | /…………/ | /…………/ | /…………/ | /………/ | /…………/ | /…………/ | /…………/ | /…………/ | /………/ | /………/ | /……/ | /…………/ |
| Chirurgiens | /…………/ | /…………/ | /…………/ | /…………/ | /…………/ | /………/ | /…………/ | /…………/ | /…………/ | /…………/ | /………/ | /………/ | /……/ | /…………/ |
| Méd. généraliste | /…………/ | /…………/ | /…………/ | /…………/ | /…………/ | /………/ | /…………/ | /…………/ | /…………/ | /…………/ | /………/ | /………/ | /……/ | /…………/ |
| Infirmier L2 | /…………/ | /…………/ | /…………/ | /…………/ | /…………/ | /………/ | /…………/ | /…………/ | /…………/ | /…………/ | /………/ | /………/ | /……/ | /…………/ |
| Infirmier A1 | /…………/ | /…………/ | /…………/ | /…………/ | /…………/ | /………/ | /…………/ | /…………/ | /…………/ | /…………/ | /………/ | /………/ | /……/ | /…………/ |
| Infirmier A2 | /…………/ | /…………/ | /…………/ | /…………/ | /…………/ | /………/ | /…………/ | /…………/ | /…………/ | /…………/ | /………/ | /………/ | /……/ | /…………/ |
| Accoucheuse L2 | /…………/ | /…………/ | /…………/ | /…………/ | /…………/ | /………/ | /…………/ | /…………/ | /…………/ | /…………/ | /………/ | /………/ | /……/ | /…………/ |
| Accoucheuse A1 | /…………/ | /…………/ | /…………/ | /…………/ | /…………/ | /………/ | /…………/ | /…………/ | /…………/ | /…………/ | /………/ | /………/ | /……/ | /…………/ |
| Accoucheuse A2 | /…………/ | /…………/ | /…………/ | /…………/ | /…………/ | /………/ | /…………/ | /…………/ | /…………/ | /…………/ | /………/ | /………/ | /……/ | /…………/ |
| Accoucheuse A3 | /…………/ | /…………/ | /…………/ | /…………/ | /…………/ | /………/ | /…………/ | /…………/ | /…………/ | /…………/ | /………/ | /………/ | /……/ | /…………/ |
| Matrone | /…………/ | /…………/ | /…………/ | /…………/ | /…………/ | /………/ | /…………/ | /…………/ | /…………/ | /…………/ | /………/ | /………/ | /……/ | /…………/ |
| Chargé de labo. | /…………/ | /…………/ | /…………/ | /…………/ | /…………/ | /………/ | /…………/ | /…………/ | /…………/ | /…………/ | /………/ | /………/ | /……/ | /…………/ |
| Chargé de pharm. | /…………/ | /…………/ | /…………/ | /…………/ | /…………/ | /………/ | /…………/ | /…………/ | /…………/ | /…………/ | /………/ | /………/ | /……/ | /…………/ |
| Autres | /…………/ | /…………/ | /…………/ | /…………/ | /…………/ | /………/ | /…………/ | /…………/ | /…………/ | /…………/ | /………/ | /………/ | /……/ | /…………/ |

**2. Prise en charge des urgences obstétricales et néonatales durant les 3 derniers mois**

| Lesquelles de ces activités les accoucheuses ont-elles effectué au cours de 3 derniers mois (précédent l’enquête) | | | *Oui=1*  *Non=2* | | Si oui, demander de voir 5 partogrammes du mois précédent et mentionner le nombre de ceux qui sont remplis. | | Si non, pourquoi  pas cette activité ?  *Manque de formation=1 ; Problème de matériel et équipement=2 ; Problème de gestion =3 ; Problème de politique sanitaire=4 ; Aucune indication=5* | |
| --- | --- | --- | --- | --- | --- | --- | --- | --- |
| **[Q21]** | Utilisation du partogramme | | /……/ | | /……………………………./ | | /……………………………………../ | |
| **[Q22]** | Administrations des antibiotiques par voie générale (**IM / IV**) | | /……/ | |  | | /……………………………………../ | |
| **[Q23]** | Affichages des indications des antibiotiques par voie parentérale (actuellement) | | /……/ | |  | | /……………………………………../ | |
| **[Q24]** | Administration des ocytociques par voie intramusculaire immédiatement après expulsion de l’enfant | | /……/ | |  | | /……………………………………../ | |
| ***[Q25]*** | Administration d’autres utérotoniques (ergométrine, méthergine, misoprostol) | | /……/ | |  | | /……………………………………../ | |
| **[Q26]** | Traction contrôlée du cordon au cours de la délivrance | | /……/ | |  | | /……………………………………../ | |
| **[Q27]** | Massage utérin après l’expulsion du placenta | | /……/ | |  | | /……………………………………../ | |
| **[Q28]** | Administration de sulfate de magnésie par voie parentérale pour traiter la pré éclampsie ou l’éclampsie | | /……/ | |  | | /……………………………………../ | |
| **[Q29]** | Administration d’autres anticonvulsivants (diazépam) pour traiter la pré éclampsie ou l’éclampsie | | /……/ | |  | | /……………………………………../ | |
| **[Q30]** | Extraction manuelle du placenta | | /……/ | |  | | /……………………………………../ | |
| **[Q31]** | Evacuation utérine (aspiration manuelle, dilatation et curetage) | | /……/ | |  | | /……………………………………../ | |
| **[Q32]** | Accouchement par voie basse avec assistance instrumentale (utilisation d’une ventouse ou forceps) | | /……/ | |  | | /……………………………………../ | |
| **[Q33]** | Transfusion sanguine | | /……/ | |  | | /……………………………………../ | |
| **[Q34]** | Césarienne | | /……/ | |  | | /……………………………………../ | |
| **[Q35]** | Prise en charge des autres urgences obstétricales | | /……/ | |  | | /……………………………………../ | |
| **[Q36]** | Administration des antibiotiques par voie orale chez les nouveau-nés | /……/ | |  | | /……………………………………../ | |  |
| **[Q37]** | Alimentation du prématuré par sonde nasogastrique | /……/ | |  | | /……………………………………../ | |  |
| **[Q38]** | Soins Kangourou aux nouveau-nés de faible poids | /……/ | |  | | /……………………………………../ | |  |
| **[Q39]** | Soins thermiques du nouveau-né à l’aide de la couveuse | /……/ | |  | | /……………………………………../ | |  |
| **[Q40]** | Oxygène chez le nouveau-né | /……/ | |  | | /……………………………………../ | |  |
| **[Q41]** | Réanimation du nouveau-né au masque (ambu) | /……/ | |  | | /……………………………………../ | |  |
| **[Q42]** | Transfusion du nouveau-né | /……/ | |  | | /……………………………………../ | |  |

**3. Référence et contre référence, permanence des services**

| **[Q43]** | **[Q44]** | **[Q45]** | **[Q46]** | **[Q47]** | **[Q48]** | |
| --- | --- | --- | --- | --- | --- | --- |
| Recours à la référence (contre référence si structure de référence)  **(3 derniers mois)**  *Oui=1*  *Non=2* | Vers quelle structure réfère-t-il (nom de la structure) | A quelle distance se trouve la structure de référence la plus proche qui prodigue des soins chirurgicaux ? | Les références de la structure de référence la plus proche sont-elles affichées ?  *Oui=1*  *Non=2* | Quels sont les moyens de transport utilisés pour la référence ?  *Ambulance=1*  *Occasion taxi=2*  *Pieds/vélo/moto=3* | Existe-t-il un roulement de garde pour les services ci-après :  *Oui=1*  *Non=2* | |
| /………../ | /……………/ | /……………..../ | /……………../ | /………….../ | Maternité | /………/ |
|  |  |  |  |  | Pharmacie | /………/ |
|  |  |  |  |  | Banque de sang | /………/ |
|  |  |  |  |  | Bloc opératoire | /………/ |
|  |  |  |  |  | Service de réanimation | /………/ |
|  |  |  |  |  | Anesthésie | /………/ |

**III. MATERIEL ET EQUIPEMENT**

|  | **Matériel et équipement** | **Nombre** | **Disponible** | **Bon état** | **Mauvais état** |
| --- | --- | --- | --- | --- | --- |
| **[Q49]** | Table d’accouchement | /………./ | /………./ | /………./ | /………./ |
| **[Q50]** | Table pour les soins néonatals | /………/ | /………/ | /………/ | /………/ |
| **[Q51]** | Table pour instruments | /………/ | /………/ | /………/ | /………/ |
| **[Q52]** | Boite à curetage | /………./ | /………./ | /………./ | /………./ |
| **[Q53]** | Boite d’accouchement | /………/ | /………/ | /………/ | /………/ |
| **[Q5]** | Bocal à pince intermédiaire | /………/ | /………/ | /………/ | /………/ |
| **[Q55]** | Balance pèse bébé | /………./ | /………./ | /………./ | /………./ |
| **[Q56]** | Tambour avec champs stérile | /………/ | /………/ | /………/ | /………/ |
| **[Q57]** | Ballon auto-gonflable avec masque N°1 | /………/ | /………/ | /………/ | /………/ |
| **[Q58]** | Table chauffante | /………./ | /………./ | /………./ | /………./ |
| **[Q59]** | Aspirateur manuel ou électrique | /………/ | /………/ | /………/ | /………/ |
| **[Q60]** | Source d’oxygène | /………/ | /………/ | /………/ | /………/ |
| **[Q61]** | Couveuse | /………./ | /………./ | /………./ | /………./ |
| **[Q62]** | Appareil de photothérapie | /………/ | /………/ | /………/ | /………/ |
| **[Q63]** | Frigo type banque de sang | /………./ | /………./ | /………./ | /………./ |
| **[Q64]** | Ventouse ou forceps | /………/ | /………/ | /………/ | /………/ |
| **[Q65]** | Tensiomètre à la salle d’accouchement | /………/ | /………/ | /………/ | /………/ |
| **[Q66]** | Foetoscope | /………./ | /………./ | /………./ | /………./ |
| **[Q67]** | Source de lumière adaptée pour l’examen gynécologique | /………/ | /………/ | /………/ | /………/ |
| **[Q68]** | Poche de sang avec trousse | /………/ | /………/ | /………/ | /………/ |
| **[Q69]** | Lit de prélèvements | /………/ | /………/ | /………/ | /………/ |

**IV. MEDICAMENTS ET REACTIFS**

|  | **Médicaments** | **Disponibles en stock** | |
| --- | --- | --- | --- |
|  |  | **Oui** | **Non** |
| **[Q70]** | Ocytocine | /………./ | /………./ |
| **[Q71]** | Sulfate de magnésie | /………/ | /………/ |
| **[Q72]** | Antibiotiques injectables | /………/ | /………/ |
| **[Q73]** | Produits sanguins | /………./ | /………./ |
| **[Q74]** | Les 4 marqueurs de la sécurité transfusionnelle | /………/ | /………/ |
| **[Q75]** | Catheter d’aspiration 8F, 10F, 12F | /………/ | /………/ |
| **[Q76]** | Sonde nasograstrique | /………./ | /………./ |
| **[Q77]** | Epicranienne | /………/ | /………/ |
| **[Q78]** | Seringue (1ml, 2 ml, 5 ml, 10ml) | /………/ | /………/ |
| **[Q79]** | Solutés à perfuser | /………./ | /………./ |

**V. OUTILS DE GESTION**

|  |  | **Utilisation**  *Oui=1 ; Non=2* | **Disponibles en stock dans le service** | |
| --- | --- | --- | --- | --- |
| **[Q80]** | **Outils de gestion** |  | **Oui** | **Non** |
| **A** | Registre CPN | /………/ | /………/ | /………/ |
| **B** | Partogramme | /………/ | /………/ | /………/ |
| **C** | Registre de maternité | /………/ | /………/ | /………/ |
| **D** | Registre des césarisées | /………/ | /………/ | /………/ |
| **E** | Fiche technique SONU | /………./ | /………./ | /………./ |
| **F** | Ordinogramme SONU | /………/ | /………/ | /………/ |
| **G** | Fiches du nouveau-né | /………/ | /………/ | /………/ |
| **H** | Registre des nouveau-nés malades | /………/ | /………/ | /………/ |
| **I** | Fiche audit des décès maternels | /………/ | /………/ | /………/ |
| **J** | Fiches d’audit des décès néonatals | /………/ | /………/ | /………/ |
| **K** | Billet de référence | /………/ | /………/ | /………/ |
| **L** | Registre CPON | /………/ | /………/ | /………/ |
| **M** | Fiche d’hémovigilance | /………/ | /………/ | /………/ |
| **N** | Bon de demande des produits sanguins | /………/ | /………/ | /………/ |
| **O** | Fiches de gestion de stock des produits sanguins | /………/ | /………/ | /………/ |

**2. Qualité des données de registres**

| **[Q81]** | Outils | Toutes les colonnes du registre  sont-elles remplies ? | | | Les données sont-elles à jour ? | | |
| --- | --- | --- | --- | --- | --- | --- | --- |
|  |  | Oui | Non | Info non disponible | Oui | Non | Info non disponible |
| **A** | Registre de la salle d’accouchement ? | 1 | 0 | 9 | 1 | 0 | 9 |
| **B** | Registre des avortements? | 1 | 0 | 9 | 1 | 0 | 9 |
| **C** | Registre du bloc opératoire ? | 1 | 0 | 9 | 1 | 0 | 9 |
| **D** | Registre de maternité | 1 | 0 | 9 | 1 | 0 | 9 |
| **F** | Registre des césarisées | 1 | 0 | 9 | 1 | 0 | 9 |
| **G** | Registre des nouveau-nés malades | 1 | 0 | 9 | 1 | 0 | 9 |
| **h** | Registre des CPON | 1 | 0 | 9 | 1 | 0 | 9 |

**VI. UTILISATION DES SERVICES**

**1. Informations sur les urgences obstétricales et néonatales en 20…**

| **[Q81]. Indicateurs** | **janvier** | **février** | **mars** | **avril** | **mai** | **juin** | **juillet** | **aout** | **Septemb.** | **octobre** | **Novem.** | **Décem.** | **Total** |
| --- | --- | --- | --- | --- | --- | --- | --- | --- | --- | --- | --- | --- | --- |
| Nouveaux cas CPN (CPN1) | /………/ | /………/ | /………/ | /………/ | /………/ | /………/ | /………/ | /……/ | /…………/ | /………/ | /………/ | /……… | /…………/ |
| Nombre des femmes CPN4 | /………/ | /………/ | /………/ | /………/ | /………/ | /………/ | /………/ | /……/ | /…………/ | /………/ | /………/ | /……… | /…………/ |
| Nombre des femmes conseillées au VIH | /………/ | /………/ | /………/ | /………/ | /………/ | /………/ | /………/ | /……/ | /…………/ | /………/ | /………/ | /……… | /…………/ |
| Nombre des femmes testées au VIH | /………/ | /………/ | /………/ | /………/ | /………/ | /………/ | /………/ | /……/ | /…………/ | /………/ | /………/ | /……… | /…………/ |
| Nombre des nouveaux cas CPON | /………/ | /………/ | /………/ | /………/ | /………/ | /………/ | /………/ | /……/ | /…………/ | /………/ | /………/ | /……… | /…………/ |
| Nombre d’accouchées | /………/ | /………/ | /………/ | /………/ | /………/ | /………/ | /………/ | /……/ | /…………/ | /………/ | /………/ | /……… | /…………/ |
| Nombre des naissances vivantes | /………/ | /………/ | /………/ | /………/ | /………/ | /………/ | /………/ | /……/ | /…………/ | /………/ | /………/ | /………/ | /…………/ |
| Nombre de césarienne | /………/ | /………/ | /………/ | /………/ | /………/ | /………/ | /………/ | /……/ | /…………/ | /………/ | /………/ | /………/ | /…………/ |
| Nombre des nouveau-nés transfusés | /………/ | /………/ | /………/ | /………/ | /………/ | /………/ | /………/ | /……/ | /…………/ | /………/ | /………/ | /……… | /…………/ |
| Femmes enceintes transfusées selon le protocole | /………/ | /………/ | /………/ | /………/ | /………/ | /………/ | /………/ | /……/ | /…………/ | /………/ | /………/ | /……… | /…………/ |
| Accouchées transfusées selon le protocole | /………/ | /………/ | /………/ | /………/ | /………/ | /………/ | /………/ | /……/ | /…………/ | /………/ | /………/ | /……… | /…………/ |
| **[Q82]. Complications de l’accouchement** |  |  |  |  |  |  |  |  |  |  |  |  |  |
| Hémorragie (ante et post-partum) | /………/ | /………/ | /………/ | /………/ | /………/ | /………/ | /………/ | /……/ | /…………/ | /………/ | /………/ | /……… | /…………/ |
| Dystocie d’obstacle ou travail prolongé | /………/ | /………/ | /………/ | /………/ | /………/ | /………/ | /………/ | /……/ | /…………/ | /………/ | /………/ | /……… | /…………/ |
| Ruptures utérines | /………/ | /………/ | /………/ | /………/ | /………/ | /………/ | /………/ | /……/ | /…………/ | /………/ | /………/ | /……… | /…………/ |
| Infection du postpartum | /………/ | /………/ | /………/ | /………/ | /………/ | /………/ | /………/ | /……/ | /…………/ | /………/ | /………/ | /……… | /…………/ |
| Pré-éclampsie sévère ou éclampsie | /………/ | /………/ | /………/ | /………/ | /………/ | /………/ | /………/ | /……/ | /…………/ | /………/ | /………/ | /……… | /…………/ |
| Avortement compliqué (hémorragie ou infection) | /………/ | /………/ | /………/ | /………/ | /………/ | /………/ | /………/ | /……/ | /…………/ | /………/ | /………/ | /……… | /…………/ |
| Nombre des cas de grossesse ectopique | /………/ | /………/ | /………/ | /………/ | /………/ | /………/ | /………/ | /……/ | /…………/ | /………/ | /………/ | /……… | /…………/ |
| Complications obstétricales indirectes | /………/ | /………/ | /………/ | /………/ | /………/ | /………/ | /………/ | /……/ | /…………/ | /………/ | /………/ | /………/ | /…………/ |

|  | **janvier** | **février** | **mars** | **avril** | **mai** | **juin** | **juillet** | **aout** | **Septemb.** | **octobre** | **Novem.** | **Décem.** | **Total** |
| --- | --- | --- | --- | --- | --- | --- | --- | --- | --- | --- | --- | --- | --- |
| **[Q83]. Décès maternels** | /………/ | /………/ | /………/ | /………/ | /………/ | /………/ | /………/ | /……/ | /…………/ | /………/ | /………/ | /……… | /…………/ |
| **[Q84]. Causes de décès maternels** |  |  |  |  |  |  |  |  |  |  |  |  |  |
| Hémorragie (ante et post-partum) | /………/ | /………/ | /………/ | /………/ | /………/ | /………/ | /………/ | /……/ | /…………/ | /………/ | /………/ | /……… | /…………/ |
| Dystocie d’obstacle ou travail prolongé | /………/ | /………/ | /………/ | /………/ | /………/ | /………/ | /………/ | /……/ | /…………/ | /………/ | /………/ | /……… | /…………/ |
| Rupture utérine | /………/ | /………/ | /………/ | /………/ | /………/ | /………/ | /………/ | /……/ | /…………/ | /………/ | /………/ | /……… | /…………/ |
| Infection du postpartum | /………/ | /………/ | /………/ | /………/ | /………/ | /………/ | /………/ | /……/ | /…………/ | /………/ | /………/ | /……… | /…………/ |
| Pré éclampsie sévère ou Eclampsie | /………/ | /………/ | /………/ | /………/ | /………/ | /………/ | /………/ | /……/ | /…………/ | /………/ | /………/ | /……… | /…………/ |
| Avortement compliqué (hémorragie ou infection) | /………/ | /………/ | /………/ | /………/ | /………/ | /………/ | /………/ | /……/ | /…………/ | /………/ | /………/ | /……… | /…………/ |
| Grossesse ectopique | /………/ | /………/ | /………/ | /………/ | /………/ | /………/ | /………/ | /……/ | /…………/ | /………/ | /………/ | /……… | /…………/ |
| Décès par causes indirectes (à spécifier ci-dessous) | /………/ | /………/ | /………/ | /………/ | /………/ | /………/ | /………/ | /……/ | /…………/ | /………/ | /………/ | /……… | /…………/ |
| **[Q85]. Décès fœtal et néonatal** |  |  |  |  |  |  |  |  |  |  |  |  |  |
| Asphyxie du nouveau-né | /………/ | /………/ | /………/ | /………/ | /………/ | /………/ | /………/ | /……/ | /…………/ | /………/ | /………/ | /……… | /…………/ |
| Prématurité (faible poids de naissance <2500g) | /………/ | /………/ | /………/ | /………/ | /………/ | /………/ | /………/ | /……/ | /…………/ | /………/ | /………/ | /……… | /…………/ |
| Infection néonatale | /………/ | /………/ | /………/ | /………/ | /………/ | /………/ | /………/ | /……/ | /…………/ | /………/ | /………/ | /……… | /…………/ |
| Malformation congénitale | /………/ | /………/ | /………/ | /………/ | /………/ | /………/ | /………/ | /……/ | /…………/ | /………/ | /………/ | /……… | /…………/ |
| Nombre des mort-nés | /………/ | /………/ | /………/ | /………/ | /………/ | /………/ | /………/ | /……/ | /…………/ | /………/ | /………/ | /……… | /…………/ |
| *Mort-nés macérés* | /………/ | /………/ | /………/ | /………/ | /………/ | /………/ | /………/ | /……/ | /…………/ | /………/ | /………/ | /……… | /…………/ |
| *Mort-nés frais* | /………/ | /………/ | /………/ | /………/ | /………/ | /………/ | /………/ | /……/ | /…………/ | /………/ | /………/ | /……… | /…………/ |
| Nombre de décès néonatales>2500g | /………/ | /………/ | /………/ | /………/ | /………/ | /………/ | /………/ | /……/ | /…………/ | /………/ | /………/ | /……… | /………/ |
| *<24 heures* | /………/ | /………/ | /………/ | /………/ | /………/ | /………/ | /………/ | /……/ | /…………/ | /………/ | /………/ | /……… | /………/ |
| *≥24 heures* | /………/ | /………/ | /………/ | /………/ | /………/ | /………/ | /………/ | /……/ | /…………/ | /………/ | /………/ | /……… | /………/ |
| Nombre de décès néonatal ≤2500g | /………/ | /………/ | /………/ | /………/ | /………/ | /………/ | /………/ | /……/ | /…………/ | /………/ | /………/ | /……… | /………/ |
